# Antibacterial Coatings on Cotton Fabrics Based on Rosmarinic Acid-Loaded Bio-Based Resin and Oxygen-Plasma Surface Pretreatment

**DOI:** 10.1021/acsomega.6c05181

**Published:** 2026-09-23

**Authors:** Norma Mallegni, Serena Facchiano, Serena Coiai, Francesca Cicogna, Roberto Spiniello, Randa Ishak, Giuseppe Rosace, Valentina Trovato, Claudia Vineis, Stefano Caporali, Elisa Passaglia

**Affiliations:** † National Research Council-Institute for the Chemistry of Organometallic Compounds (CNR-ICCOM), SS Pisa, Via Moruzzi 1, 56124 Pisa, Italy; ‡ National Research Council-Institute of Intelligent Industrial Technologies and Systems for Advanced Manufacturing (CNR-STIIMA), Corso Giuseppe Pella 16, 13900 Biella, Italy; § Department of Civil and Industrial Engineering, University of Pisa, Largo L. Lazzarino 1, 56122 Pisa, Italy; ∥ Department of Engineering and Applied Sciences, University of Bergamo, Viale Marconi 5, 24044 Dalmine, BG, Italy; ⊥ Department of Industrial Engineering, DIEF, University of Florence, Via S. Marta 3, 50139 Florence, Italy

## Abstract

The development of antibacterial cotton textiles is of considerable interest for applications in healthcare, hygiene, and protective materials, where limiting microbial proliferation on fibrous surfaces is essential. However, many of the finishing strategies currently reported for cotton rely on metal-based agents or multifunctional synthetic systems, which may raise concerns about environmental impact, uncontrolled release, and long-term durability. In this context, the use of naturally derived bioactive compounds, combined with surface activation methods, represents a promising route toward more sustainable antimicrobial coatings. In this study, cotton fabrics were functionalized with a commercial biobased acrylic resin that contained increasing amounts of rosmarinic acid, a plant-derived polyphenol with recognized antimicrobial activity. The effect of oxygen plasma pretreatment was investigated to assess whether surface activation could improve coating distribution, interfacial adhesion, and functional performance. Resin films and coated fabrics were characterized by infrared spectroscopy, thermogravimetric analysis, differential scanning calorimetry, and scanning electron microscopy, while antibacterial activity was evaluated against *Staphylococcus aureus* and *Escherichia coli*. The results showed that plasma pretreatment promoted a more homogeneous distribution of the coating on cotton and qualitatively supported a more effective organization of the resin-based functional layer. The incorporation of rosmarinic acid modified the thermal behavior of the resin, indicating reduced chain mobility and more importantly the occurrence of interaction with the polymer matrix. The coated fabrics displayed marked antibacterial activity against *S. aureus*, whereas the effect against *E. coli* was more strongly dependent on rosmarinic acid content and coating uniformity. Overall, the study demonstrates that combining plasma surface activation with a biobased resin containing rosmarinic acid is a viable strategy for preparing metal-free antibacterial cotton coatings.

## Graphical abstract

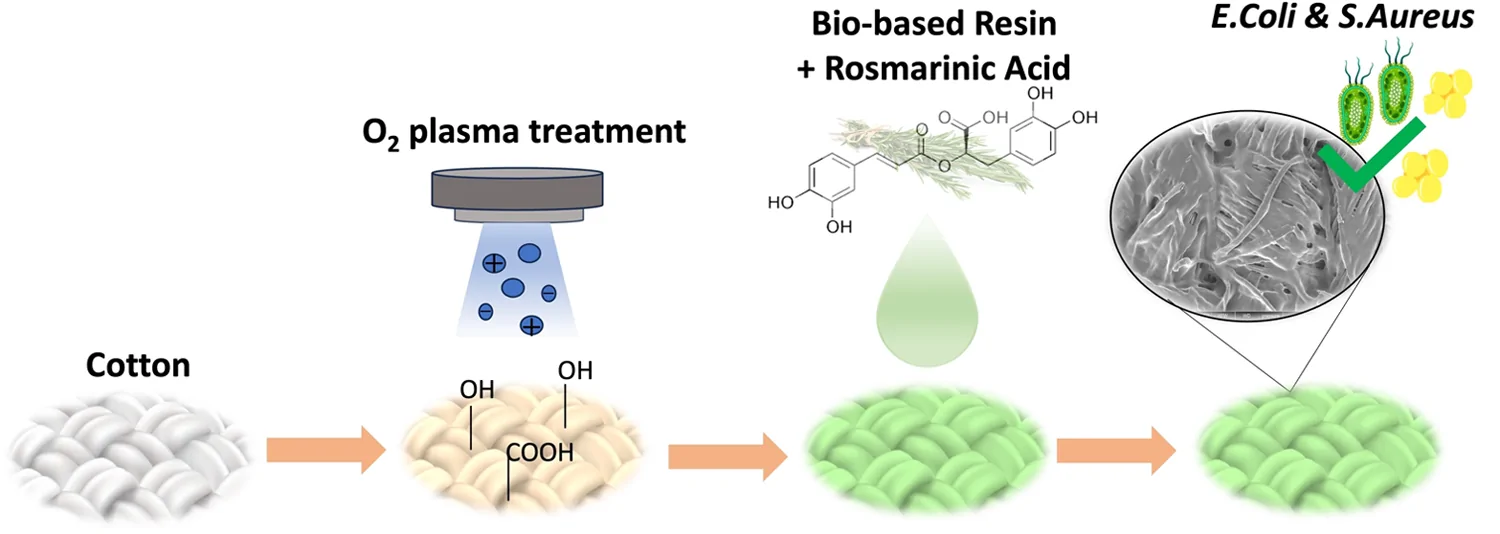

## 1 Introduction

Antibacterial textiles are attracting increasing attention for healthcare, hygiene, and protective applications, where limiting microbial proliferation on fibrous surfaces is important not only for hygienic reasons, but also for material durability and user safety.^1–3^ Cotton is one of the most relevant substrates for these applications because it combines comfort-related air and moisture-management properties with broad use in medical and hygienic products, including surgical clothing, surgical covers, bedding, masks, and wound-contact materials.^4,5^ At the same time, cotton does not provide intrinsic and durable antibacterial protection, and the stable immobilization of bioactive agents on cellulose is often limited by interfacial affinity, coating heterogeneity, and insufficient resistance to washing or use conditions.^1,2^ Among the antibacterial finishing, the approaches most frequently reported in the textile literature are metal- and metal oxide-based systems, cationic organic agents, N-halamine derivatives, biopolymer-based treatments, sol–gel hybrid coatings, and, more recently, naturally derived low-molecular-weight compounds. A review published in early 2013 identified triclosan, silane quaternary ammonium compounds, zinc pyrithione, and silver-based compounds as the main classes of antimicrobial agents used to functionalize textiles.^6^ Since then, the field has broadened considerably. Metal- and metal oxide-based systems, including silver, copper, zinc oxide, and titanium dioxide, remain widely investigated because of their broad-spectrum efficacy, although concerns about leaching, long-term exposure, and environmental impact persist.^7^ Within this area, recent hybrid sol–gel approaches have described the incorporation of silver nanoparticles into organically modified silica xerogels, which are subsequently dispersed in partly biobased textile finishing resins, to obtain antibacterial cotton coatings with improved physical and chemical stability and effective deposition by rod-coating.^8^ However, these systems still rely on silver as the active antimicrobial component. Cationic organic finishes, particularly quaternary ammonium-based systems, continue to represent an important class because they can provide strong contact-active and durable antibacterial effects when suitably anchored to cotton.^9^ Another relevant category is represented by N-halamine chemistries, which are attractive for their high antimicrobial efficacy, broad-spectrum activity, and regenerable oxidative functionality.^10^ In parallel, biopolymer-based treatments, especially those based on chitosan, have long been investigated as more benign alternatives, although their effectiveness and durability often depend on appropriate cross-linking or fixation strategies.^11^ Sol–gel and hybrid inorganic–organic coatings have also been extensively explored as carrier matrices able to improve the deposition and retention of active species on textile substrates.^12^ More recently, increasing attention has shifted toward plant-derived phenolic compounds and other biobased antimicrobial agents, which are appealing from a sustainability perspective, but still present critical issues regarding coating homogeneity, fixation, and durability on cotton.^13^ In this context, natural organic acids have also been proposed as antibacterial finishing agents for cotton, both alone and in combination with biobased polymeric and polycarboxylic binders. Recent results on lactic acid, l-ascorbic acid, and tartaric acid showed high initial antibacterial activity, while also indicating that durability under aqueous laundering remains a key limitation, even when binder-assisted formulations are used; by contrast, some acid-binder systems showed better retention after dry-cleaning.^14^ This state-of-the-art highlights that, despite the broad range of available antibacterial finishes, the development of metal-free systems able to combine efficacy, sustainability, coating homogeneity, and sufficient retention on cotton remains an open challenge.

In parallel with the search for metal-free and environmentally friendly antibacterial agents, increasing attention has been devoted to surface-activation strategies able to improve the deposition and retention of functional coatings on cotton Plasma treatment represents a versatile technique for selective surface modification, as it increases surface reactivity and promotes the anchoring of metal- and polymer-based coatings without affecting the bulk mechanical properties of the substrate.^15–18^ Cold plasma has also been successfully employed for the in situ synthesis of silver nanoparticles on cotton fabrics without the use of chemical reducing agents, enabling uniform deposition while preserving the cellulose structure.^19,20^ In addition, green in situ synthesis of silver nanoparticles directly on cotton fabrics using plant-derived extracts as reducing agents has also been reported as an environmentally friendly strategy to produce antibacterial and UV-protective textiles.^21^

However, the use of naturally derived antimicrobial agents is increasingly considered a more sustainable alternative. In this regard, several studies have investigated plant extracts and polyphenolic compounds for textile functionalization;^13,22^ however, their practical application is often limited by poor immobilization on textile substrates, especially on cotton, and pretreatment of fibers is necessary to prevent rapid loss of activity upon washing.^23^ To address this issue, cold plasma has been used to activate polymer nonwoven fabrics prior to polyphenol immobilization, enhancing surface functionalization and improving the retention of bioactive molecules eventually anchored onto inorganic carriers.^24–26^ With a similar purpose, partially biobased commercial resins have been used as embedding matrices for SilverSil xerogels containing sol–gel-entrapped silver nanoparticles on cotton and polyester fabrics.^8^ Although this strategy further improved coating homogeneity and stability, the antimicrobial activity still relied on the presence of metal nanoparticles. Therefore, the development of metal-free antimicrobial systems with enhanced coating capability remains highly desirable. A promising approach involves the incorporation of plant-derived bioactive compounds into polymeric matrices used as coating agents.

A particularly interesting candidate within this framework is rosmarinic acid, a naturally occurring hydroxycinnamic-derived polyphenol widely found in aromatic plants, especially in the Lamiaceae family, and chemically consisting of an ester of caffeic acid and 3,4-dihydroxyphenylacetic acid.^27,28^ Rosmarinic acid has attracted considerable attention because of its documented antioxidant, antimicrobial, and antibiofilm activities,^29^ which make it a promising bioactive compound for the development of functional surfaces based on naturally derived chemistry.^29,30^

From a formulation standpoint, rosmarinic acid is also of interest because its molecular structure includes multiple phenolic hydroxyl groups, along with ester and carboxylic functionalities, which can favor secondary intermolecular interactions with polar polymer matrices. On this basis, its incorporation into an acrylic resin can be regarded as a rational strategy to combine the bioactivity of a low-molecular-weight polyphenol with the film-forming ability of a polymeric carrier phase.^31^ However, as reported more generally for phenolic compounds applied to textiles, the direct use of such molecules is often limited by nonuniform deposition, insufficient fixation to the substrate, and progressive loss of functionality during use or washing unless suitable immobilization strategies are adopted.^13,23^ In this context, combining rosmarinic acid with a biobased acrylic resin and oxygen-plasma pretreatment of cotton appears particularly relevant. The resin may serve as a continuous carrier phase for the bioactive compound, while plasma activation may improve coating spreading and interfacial adhesion on the cellulosic surface.^32,33^

Based on these considerations, the present work investigates cotton fabrics functionalized with a commercial biobased acrylic resin containing increasing amounts of rosmarinic acid, with and without prior oxygen-plasma treatment of the substrate. The study was designed to clarify the role of plasma activation in promoting coating homogeneity and to assess how these effects influence the thermal behavior and antibacterial response of the treated fabrics. Particular attention was devoted to the relationship between rosmarinic acid loading, surface distribution of the coating, and antibacterial performance against *Staphylococcus aureus* and *Escherichia coli*.

## 2 Materials and Methods

### 2.1 Materials

Test Fabrics Inc. (West Pittston, PA, USA) kindly provided the plain-woven bleached cotton fabric (hereafter referred to as C), with mass per unit area of 110 g m^–2^. Before use, the fabric was conditioned under standard laboratory conditions (20 °C and 65% Relative Humidity) and used as received, unless otherwise stated.

The commercial resin (Vision Sugar 4.0), exhibiting a dry solid content of 0.456 g/mL as determined gravimetrically (2.28 g of dry residue obtained from 5 mL of the commercial formulation), was kindly provided by F.T.R. Srl (Albano Sant’Alessandro, Italy). For the preparation of the coating solutions, 0.5 mL of the resin stock solution (corresponding to 0.228 g of dry polymer) was diluted to a final volume of 5.0 mL, yielding a final polymer concentration of 45.6 g/L.

Ethanol (ACS Reagent, 96%) and rosmarinic acid (RA, 96%) were purchased from Sigma-Aldrich. Deionized ultrapure water was obtained using a Milli-Q system (Millipore, Bedford, MA, USA).

The antibacterial activity was evaluated using model strains obtained from the American Type Culture Collection (ATCC): Gram-positive bacteria, *S. aureus* ATCC 6538 and Gram-negative bacteria, *E. coli* ATCC 11229 (supplied by Biogenetics Diagnostics Srl, Italy). The media used in the ASTM E 2149–25 “Standard Test Method for Determining the Antimicrobial Activity of Antimicrobial Agents Under Dynamic Contact Conditions” was Yeast Extract Agar (nutrient broth agar) supplied by Merck Life Science (Milano, Italy).

### 2.2 Cotton Surface Plasma Activation

Cotton fabric samples (4 cm × 4 cm) were treated with plasma using a continuous reactor (Tucano, Gambetti, Italy), featuring a working area of 118 mm × 310 mm and a chamber volume of about 5.5 L. The reactor was equipped with an aluminum electrode and powered by a 13.56 MHz radio frequency (RF) generator with a maximum output power of 200 W. Gas flow was controlled by two mass flow controllers (MFCs). The vacuum chamber was evacuated using a two-stage Edwards RV5 rotary vane vacuum pump (model A65301903), providing a nominal pumping speed of 5.1 m^3^ h^–1^ (50 Hz) (Figure S1). The treatment protocol included four main stages: (i) evacuation of the chamber from atmospheric pressure down to approximately 0.2 mbar; (ii) pressure stabilization for 5 s; (iii) introduction of the selected gas (oxygen); and (iv) activation of the generator to start the plasma process. During treatment, the pressure was kept within the range of 0.3–0.8 mbar. Samples were exposed for 5 min on a single side, followed by venting back to atmospheric pressure (around 150 s). The plasma power was set at 50 W, while the oxygen flow rate was maintained at 15 sccm. After plasma treatment, the samples were characterized by Fourier Transform Infrared Spectroscopy–Attenuated Total Reflectance (FTIR-ATR), Thermogravimetric Analysis (TGA), Scanning Electron Microscopy (SEM), and X-ray Photoelectron Spectroscopy (XPS).

### 2.3 Preparation of RA-Loaded Resin Solutions and Coating of Cotton and Plasma-Activated Cotton

All resin solutions were prepared by adding 0.5 mL of commercial resin to 4.5 mL of a water–ethanol mixture (95:5 v/v). Increasing amounts of RA were then added to obtain final concentrations of 5, 10, 15, and 20 g/L.

1 mL of each resin solution containing increasing amounts of RA (0, 5, 10, 15, and 20 g/L) was drop-cast into a Teflon Petri dish and allowed to evaporate at room temperature for approximately 1 h. The resulting samples were then placed in an oven at 100 °C for 15 min and subsequently cured at 170 °C for 3 min. Once cross-linked, the resins were removed from the substrate and characterized by FTIR-ATR, TGA, and Differential Scanning Calorimetry (DSC).

Cotton specimens, both untreated and plasma-treated, with dimensions of 4 cm × 4 cm, were dip-coated in the prepared resin solutions (5 mL each). The samples were then dried at room temperature, followed by thermal treatment at 100 °C for 15 min and at 170 °C for 3 min. All samples were characterized by FTIR-ATR, TGA, DSC, SEM and measurements focused on testing antibacterial properties. Table 1 summarizes all prepared formulations and their corresponding nomenclature used hereafter. The notation R_*x*RA refers to resin formulations prepared with × g L^–1^ of rosmarinic acid in the starting coating solution, and the same designation is retained for the corresponding cured resin films.

**Table 1:** Sample Nomenclature

sample	code
Cotton	C
Resin solution (45.6 g/L)	R
Resin solution (45.6 g/L) containing RA (5 g/L)	R_5RA
Resin solution (45.6 g/L) containing RA (10 g/L)	R_10RA
Resin solution (45.6 g/L) containing RA (15 g/L)	R_15RA
Resin solution (45.6 g/L) containing RA (20 g/L)	R_20RA
Plasma-treated cotton	P_C
Cotton dip-coated with resin solution (45.6 g/L)	C_R
Plasma-treated cotton dip-coated with resin solution (45.6 g/L)	P_C_R
Cotton dip-coated with resin solution (45.6 g/L) containing RA (5 g/L)	C_R_5RA
Plasma-treated cotton dip-coated with resin solution (45.6 g/L) containing RA (5 g/L)	P_C_R_5RA
Cotton dip-coated with resin solution (45.6 g/L) containing RA (10 g/L)	C_R_10RA
Plasma-treated cotton dip-coated with resin solution (45.6 g/L) containing RA (10 g/L)	P_C_R_10RA
Cotton dip-coated with resin solution (45.6 g/L) containing RA (15 g/L)	C_R_15RA
Plasma-treated cotton dip-coated with resin solution (45.6 g/L) containing RA (15 g/L)	P_C_R_15RA
Cotton dip-coated with resin solution (45.6 g/L) containing RA (20 g/L)	C_R_20RA
Plasma-treated cotton dip-coated with resin solution (45.6 g/L) containing RA (20 g/L)	P_C_R_20RA

### 2.4 Samples Characterization

The infrared spectra of all samples were recorded using a PerkinElmer Spectrum Two FTIR spectrophotometer (Norwalk, CT, USA) equipped with a diamond crystal attenuated total reflectance (ATR) accessory. Each spectrum was obtained by averaging 32 scans at a resolution of 4 cm^–1^.

For cotton samples, a preliminary FTIR-ATR analysis was performed on the untreated cotton surface to establish a rigorous plasma treatment protocol. At least five points on the sample surface (Figure S2I) were analyzed to ensure measurement reproducibility. Both sides of the commercial cotton, labeled A and B, were examined to assess possible differences in surface composition (Figure S2II). The peak observed at 1540 cm^–1^ in side B is generally associated with the −COO^–^ stretching mode, which in cotton can be attributed to the presence of sodium stearate, commonly used to enhance fabric hydrophobicity or confer self-cleaning properties.^34,35^ After plasma treatment, which also exerts a chemical-physical cleaning effect on the surface, this peak tends to disappear, indicating the removal of sodium stearate. To ensure uniformity and comparability, side A was consistently selected for all treatments and analyses.

Thermogravimetric analysis (TGA) was performed using a Seiko EXSTAR 7200 TGA/DTA Instruments (Masterlab, Milan, Italy). The onset temperature (*T*_onset_) was determined by both the tangent intersection method and by considering the temperature corresponding to a 5% mass loss. Approximately 5–10 mg of each sample was analyzed in triplicate under a nitrogen flow of 200 mL·min^–1^, with the temperature increased from 30 to 700 °C at a heating rate of 10 °C·min^–1^.

Differential scanning calorimetry (DSC) was carried out using a PerkinElmer DSC-4000 thermal analyzer (PerkinElmer, Waltham, MA, USA), equipped with an intracooler and calibrated with zinc (melting point: 419.5 °C) and indium (melting point: 156.6 °C, Δ*H* = 28.5 J·g^–1^). Samples (5–10 mg) underwent the following thermal cycle: heating from 30 to 200 °C at 10 °C·min^–1^ and holding for 3 min; cooling from 200 to 30 °C at 10 °C·min^–1^ and holding for 3 min; followed by a second heating from 30 to 200 °C at 10 °C·min^–1^. Glass transition temperatures (*T*_g_) were determined from the second heating run using Pyris V9.0 software.

Scanning electron microscopy (SEM) was performed at the Centro per l’Integrazione della Strumentazione Scientifica, Università di Pisa (CISUP), using an FEI Quanta 450 FEG-SEM (ThermoFisher Scientific, Pardubice, Czech Republic) equipped with a Bruker QUANTAX XFlash 6|10 EDX spectrometer (Berlin, Germany). Samples were coated with a 10 nm platinum layer to provide a conductive surface. SEM micrographs were acquired in secondary electron (SE) mode using a Large Field Detector (LFD) at an accelerating voltage of 10 kV. The working distance was approximately 8–9 mm.

XPS measurements were performed using an ultrahigh-vacuum (UHV) system (base pressure ∼ 10^–9^ mbar) equipped with a VSW HAC 500 hemispherical electron energy analyzer (HA100, VSW Scientific Instruments Ltd., Manchester, UK) and a nonmonochromated Mg Kα X-ray source (1254 eV, TA10) operated at 12 kV and 10 mA (120 W). Samples were mounted on the sample holder using conductive carbon tape and transferred into the analysis chamber under a nitrogen atmosphere to minimize surface contamination. Before analysis, the specimens were kept in the introduction chamber for approximately 24 h. Survey spectra and high-resolution core-level spectra were recorded in constant analyzer energy (CAE) mode using a pass energy of 22 eV, with energy step sizes of 1.0 and 0.1 eV, respectively. Spectral processing and peak deconvolution were carried out using CasaXPS software after Shirley background subtraction and peak fitting with mixed Gaussian–Lorentzian functions. The binding energy scale was calibrated by referencing the aliphatic C 1s peak to 284.8 ± 0.1 eV.

Antibacterial tests were performed by following the standard methodology ASTM E2149–25 “Standard test method for determining the antimicrobial activity of antimicrobial agents under dynamic contact conditions” against *S. aureus* ATCC 6538 (Gram positive) and *E. coli* ATCC 11229 (Gram negative). The tests were carried out by diluting the bacterial inoculum up to 1.5–3.0 × 10^5^ CFU mL^–1^. About 1 g of the sample was immersed in 50 mL of inoculum and stirred (190 rpm) for 1 h at room temperature. After that, 1 mL of solution was incubated for 24 h in Yeast Extract Agar at 37 °C. The tests were performed in duplicate. Although triplicate testing is generally recommended, the duplicate measurements showed good repeatability, as confirmed by the low variability observed in the results reported in Table 4. All reported values represent the average of independent measurements, and the variability between replicates remained limited under the adopted experimental conditions. Nevertheless, this represents a limitation of the present study, and the antibacterial results should therefore be interpreted as preliminary, particularly for samples showing low bacterial reduction and greater variability. The antibacterial activity was expressed as bacterial reduction (%) and calculated according to eq 1

1 $$b\mathrm{acteria}\;\mathrm{reduction}\;\left(\%\right)=\frac{\left(B-A\right)}{B}\times 100$$

where *A* is the number of bacterial colonies in the diluted inoculum in contact with the sample, and *B* is the number of bacterial colonies in the diluted inoculum.

## 3 Results and Discussion

### 3.1 Characterization of Cotton (C) and Plasma-Treated Cotton (P_C)

The FTIR-ATR spectra of untreated and plasma-treated cotton are shown in Figure S3. The broad band around 3330 cm^–1^ was assigned to the stretching vibrations of −OH groups in cellulose. The signal at 2904 cm^–1^ is attributed to the stretching vibrations of C–H bonds characteristic of cellulose and hemicellulose. The band at 1431 cm^–1^ is associated with the symmetric bending of CH_2_ groups in cellulose, while the bands at 1370 and 1315 cm^–1^ are assigned to the bending vibrations of C–H and C–O bonds, respectively, within the polysaccharide rings of cellulose. The intense band at 1031 cm^–1^ arises from the stretching vibrations of C–O and O–H groups in polysaccharides, whereas the peak at 893 cm^–1^ indicates the presence of β-glycosidic linkages between the monosaccharide units of cellulose.^36,37^ Only minor differences were observed between the spectra of untreated and plasma-treated cotton. In particular, an increased absorption in the 1750–1660 cm^–1^ range and in the appearance of a band near 1730 cm^–1^, which can be attributed to the C=O stretching vibrations of carboxyl and carbonyl functionalities, can be observed^16,38,39^ (Figure S3). Since oxygen plasma modifies only the outermost surface of the fibers, whereas the penetration depth of FTIR-ATR extends well beyond the modified layer, the surface chemical modifications were further investigated by XPS, (Figure 1).

**Figure 1:**
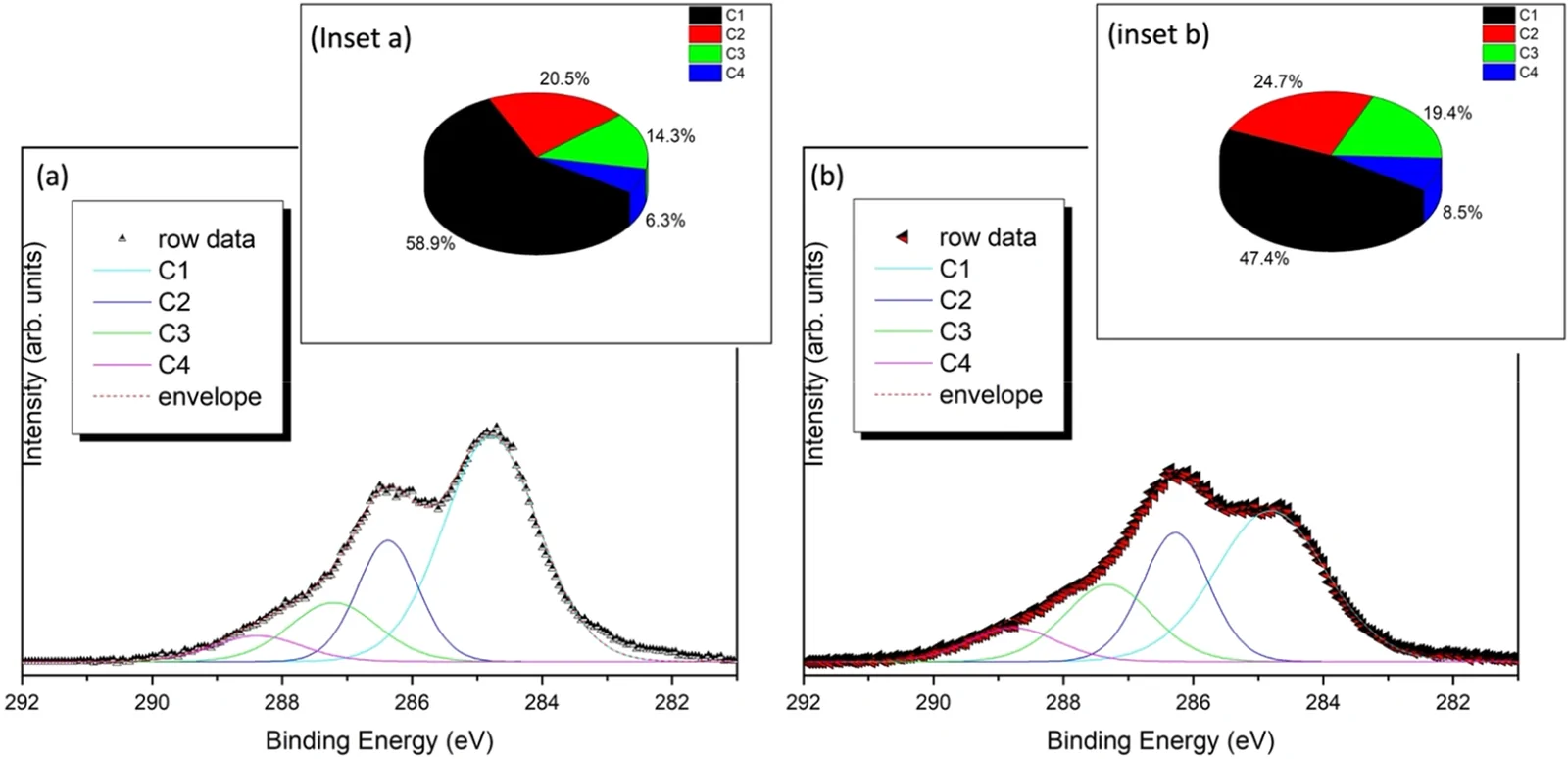
XPS spectra of (a) untreated cotton and (b) plasma-treated cotton, together with the corresponding C 1s peak deconvolution. The fitted components are assigned as follows: C1, C–C/C–H; C2, C–O; C3, C=O; and C4, O–C=O. Insets (a) and (b) show the relative percentages of the fitted C 1s components (C1–C4) for untreated and plasma-treated cotton, respectively.

The XPS survey spectra confirmed that carbon and oxygen were the predominant elements detected on the fiber surface, while no additional elements were observed above the instrumental detection limit (Figure S4). Although the O 1s spectra showed only limited variations after plasma treatment, the high-resolution C 1s spectra provided clear evidence of surface oxidation. Deconvolution of the C 1s envelope into four components corresponding to carbon atoms with progressively higher oxidation states showed that C1, centered at approximately 284.8 eV, was assigned to aliphatic C–C/C–H carbon; C2, at approximately 286.3 eV, to C–O; C3, at approximately 287.3 eV, to more oxidized carbon environments associated with C=O species; and C4, at approximately 288.4–288.8 eV, to O–C=O functionalities, including carboxylic and ester groups. The relative contribution of C1 decreased after plasma treatment, whereas the contributions of the oxygen-containing C2–C4 components increased (Figure 1). In addition, the total content of oxygen increased from 26.4% for C to 41.1% for P_C. These results confirm that oxygen plasma promotes oxidation of the outermost cotton surface through the introduction of oxygen-containing functional groups.^40^

SEM observations reveal that plasma treatment induces distinct surface indentations, increasing the roughness of the fibers (Figure 2). In particular, Figure 2a, corresponding to untreated cotton, shows the typical longitudinal fibril structure characterized by a smooth surface free of roughness, as well as the grooved morphology of cotton. In contrast, Figure 2b, which was collected after 5 min of plasma treatment, shows a rougher fiber surface, indicating an etching effect.^40^

**Figure 2:**
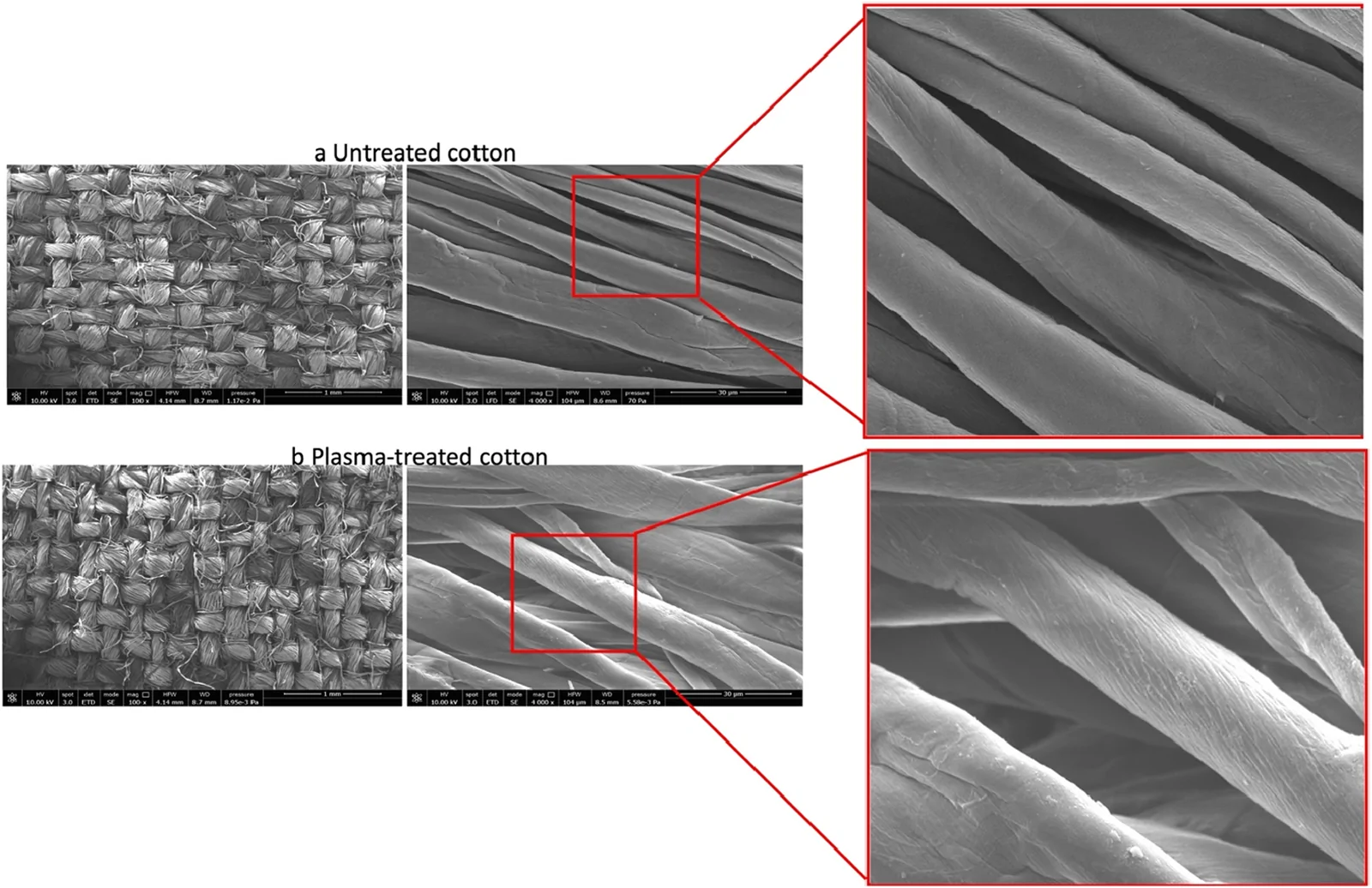
SEM analysis at different magnifications (100× and 4000×) of cotton and plasma-treated cotton. On the right, details of both untreated cotton (C) and plasma-treated cotton (P_C) fibers are highlighted in the boxed areas, showing the smoother surface of the untreated fibers and the rougher surface after plasma treatment. Magnification is provided for qualitative observation only.

Thermogravimetric analyses (TGA) were carried out under a nitrogen atmosphere to evaluate the thermal stability of cotton before and after plasma treatment (Figure S5). Both thermogravimetric (TG) and first derivative (DTG) curves are shown. An initial weight loss in the temperature range of 40–200 °C, corresponding to approximately 4% and 3% of the sample mass for C and P_C, respectively, was observed and can be attributed to the evaporation of physically adsorbed moisture.

Cotton fibers are predominantly composed of cellulose, with minor contributions from hemicellulose and lignin.^36^ These components thermally decompose over partially overlapping temperature ranges: hemicellulose typically degrades between 240 and 310 °C, cellulose between 310 and 360 °C, and lignin over a broader range, approximately 200–550 °C.^41^ Although lignin degradation may extend to higher temperatures, its contribution in cotton fibers is limited due to its low content.^36,41^ Under a nitrogen atmosphere, both untreated and plasma-treated cotton samples exhibit a single main degradation step, mainly associated with cellulose decomposition. The onset degradation temperature (*T*_onset_), defined as the temperature corresponding to 5% mass loss, is approximately 296 °C for untreated cotton and 308 °C for plasma-treated cotton, indicating a slight delay in the onset of thermal degradation following plasma treatment. In contrast, the maximum degradation temperature (*T*_max_ in DTG) remains nearly unchanged at around 370 °C for both samples, suggesting that the primary degradation mechanism of cellulose is preserved. Overall, these results indicate that plasma treatment slightly improves the thermal stability of cotton without significantly affecting its bulk thermal degradation behavior. The observed increase in *T*_onset_ is consistent with literature reports indicating that cold plasma treatments, including oxygen-based plasmas, may influence the thermomechanical features of cellulosic materials.^42,43^ Such effects can be attributed to surface oxidation and possible physical or chemical cross-linking,^44^ which can modify the initial degradation behavior, owing to increased interaction between fibers generating also stronger packing. In addition, differences in moisture content (higher in case of sample C) can also affect the *T*_onset_ value.

### 3.2 Characterization of Rosmarinic Acid-Loaded Resins (R_*x*RA with *x* = 5,10,15,20)

As described in the experimental section, aliquots of each resin solution containing increasing amounts of RA (0, 5, 10, 15, and 20 g/L) were drop-cast and allowed to evaporate to remove water. The resulting films were subsequently cured and characterized by FTIR-ATR, TGA, and DSC.

#### 3.2.1 FTIR-ATR Characterization

FTIR-ATR spectra of pure resin (R), pure rosmarinic acid (RA), and RA-loaded resins (R_*x*RA) were collected and compared (Figure 3a,3b). The FTIR-ATR spectrum of pure RA shows a broad absorption band in the 3650–3200 cm^–1^ region, attributed to O–H stretching vibrations of phenolic and carboxylic groups. The band in the 3000–2900 cm^–1^ region is assigned to C–H stretching vibrations of aromatic carbon atoms. A characteristic absorption at 1718 cm^–1^ corresponds to the C=O stretching vibration of the carboxylic acid group, while the band at 1650 cm^–1^ is attributed to the C=O stretching of conjugated carboxylic groups and is likely affected by hydrogen bonding. This interpretation is in agreement with literature data reported for isolated RA.^45^ The peak at 1605 cm^–1^ is associated with C=C stretching vibrations of the aromatic ring, with additional bands observed at 1610 and in the 1516–1465 cm^–1^ range. Furthermore, the bands between 1270 and 1020 cm^–1^ are assigned to C–O and C–O–C stretching vibrations of phenolic and ester groups, in agreement with literature data^45–47^

**Figure 3:**
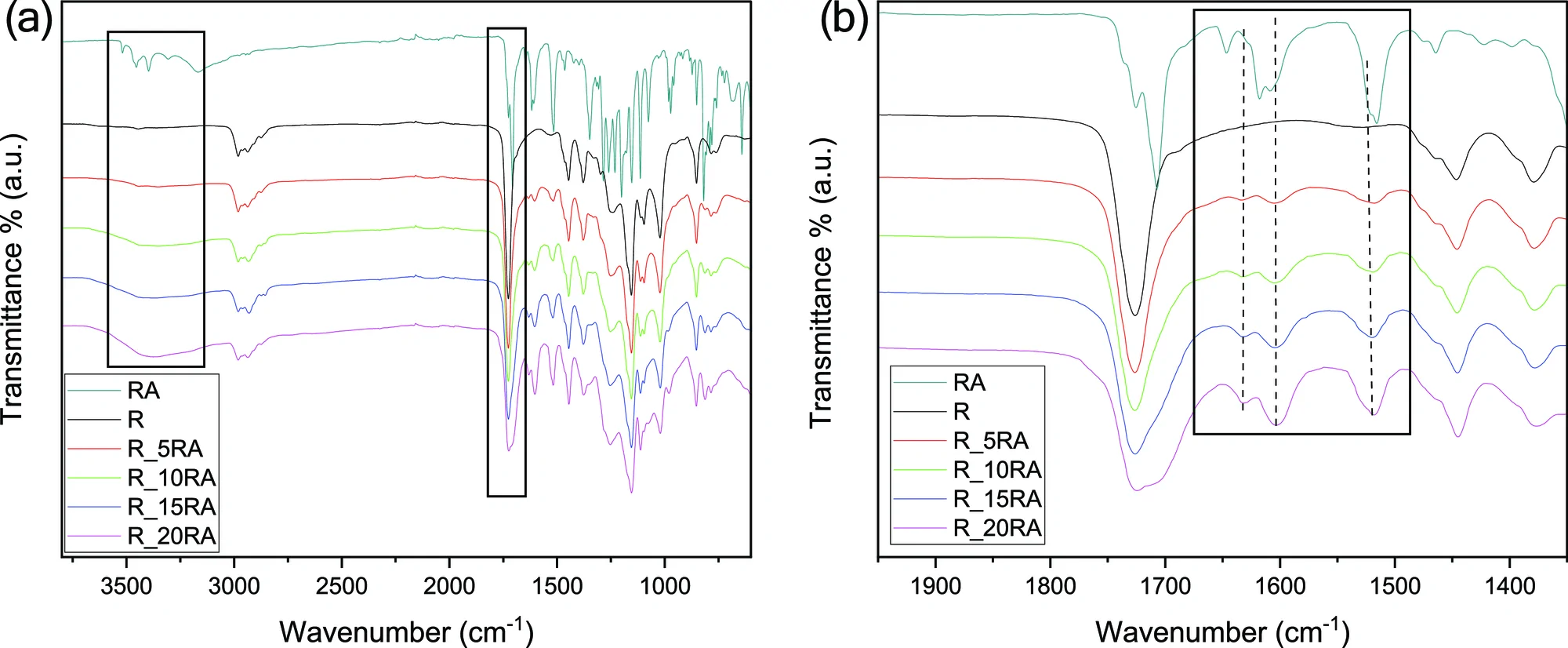
FTIR-ATR spectra of pure R, RA and R samples containing increasing amounts of RA (5, 10, 15, 20 g/L) (a) full spectra and (b) magnified view of the 1950–1400 cm*^–^* ^1^ spectral region.

The pure resin exhibits a FTIR-ATR spectrum typical of acrylic-based materials, closely resembling that of methacrylate resins. The band at approximately 2940 cm^–1^ is attributed to aliphatic C–H stretching vibrations. A strong absorption at around 1729 cm^–1^ corresponds to the ester carbonyl (C=O) stretching vibration, which is characteristic of acrylic systems. The peak at 1446 cm^–1^ is assigned to C–H bending vibrations, while the band at 1247 cm^–1^ is related to C–C–O stretching of ester groups. The absorption at approximately 1155 cm^–1^ is attributed to asymmetric C–O–C stretching, and the bands at 853 and 777 cm^–1^ are associated with CH_2_ rocking modes.

The spectra of RA-loaded resins display contributions from both the polymer matrix and RA. Increasing RA content results in a progressive enhancement of its characteristic bands: O–H stretching vibrations (Figure 3a) and the signals in the 1650–1450 cm^–1^ region (Figure 3b), which are mainly associated with the aromatic structure of RA. In addition, a broadening and partial overlap of the C=O stretching region is observed, likely due to the contribution of both the ester groups of the resin and the carboxylic functionalities of RA. The bands at 1632–1606 and 1518 cm^–1^, attributed to aromatic C=C stretching vibrations, exhibit slight shifts toward lower and higher wavenumbers (as evidenced in the box of Figure 3b), suggesting the possible occurrence of intermolecular interactions between RA and the resin matrix. These spectral changes, including band shifts and intensity variations, are consistent with noncovalent interactions, primarily hydrogen bonding, between the hydroxyl and carboxylic groups of RA and the carbonyl groups of the acrylic resin.^48^

Furthermore, no new absorption bands clearly attributable to reaction products between RA and the resin were observed. Accordingly, the FTIR-ATR results mainly suggest the physical incorporation of RA into the polymer matrix, possibly accompanied by secondary intermolecular interactions.

#### 3.2.2 Thermal Characterization (TGA and DSC)

The TGA thermograms, shown in Figure 4 for all samples with increasing concentrations of RA, clearly reflect the resin compositions. RA exhibits a two-step degradation process, with a first decomposition event around 225 °C and a second at higher temperatures, approximately 365 °C. In contrast, the neat resin shows a one-step degradation pattern with *T*_onset_ and *T*_max_ at about 341 and 405 °C, respectively. As the RA concentration increases, the decomposition curve shifts progressively toward lower temperatures, indicating greater susceptibility of the material to thermal degradation (Figure 4a).

**Figure 4:**
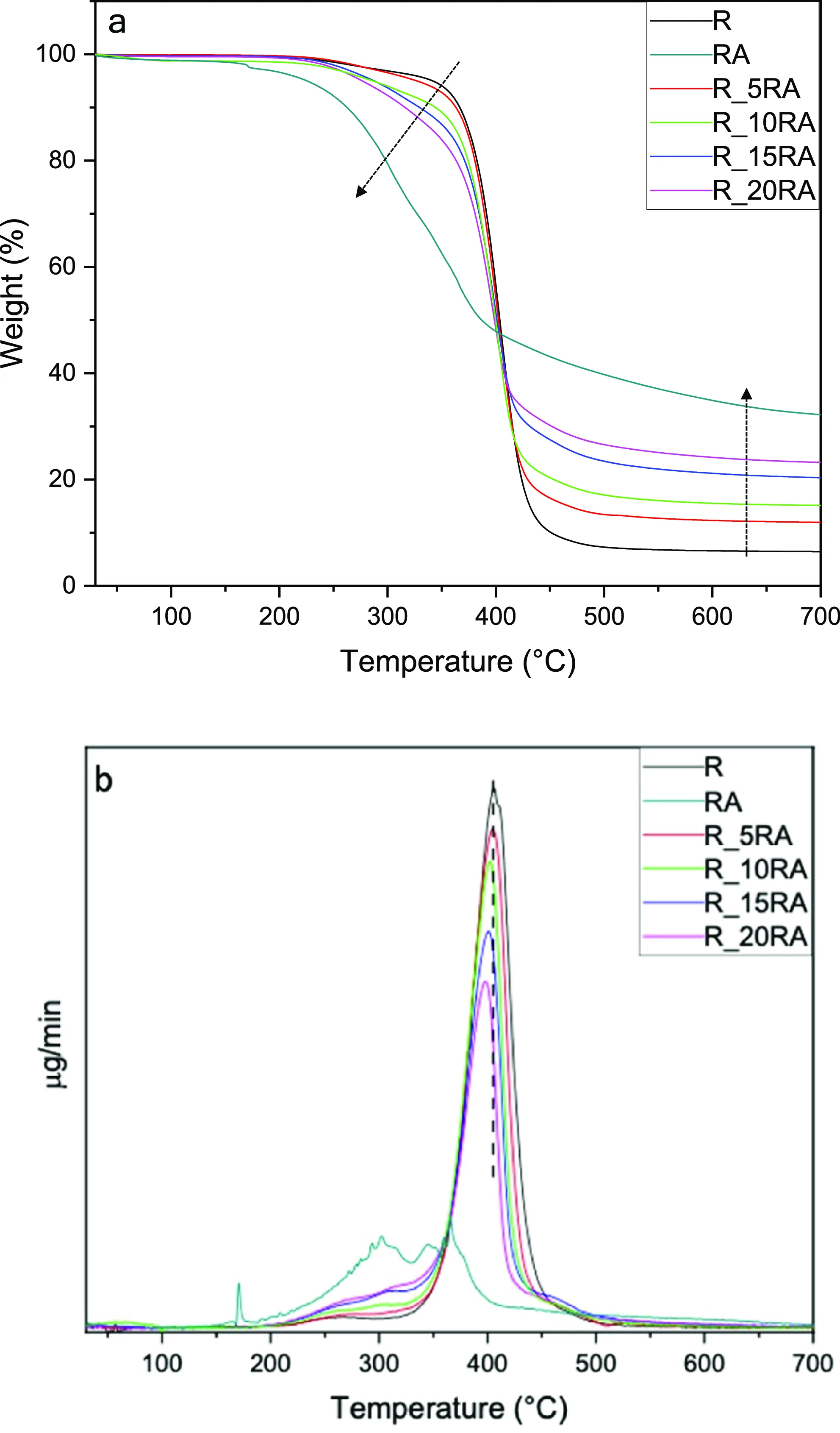
Thermogravimetric analysis (TGA) of the resin containing increasing concentration of RA (5, 10, 15, and 20 g/L): (a) TG, and (b) (DTG) curves.

This trend is quantitatively reflected by the progressive decrease of *T*_max_ in the DTG curves, from 405 °C for the neat resin (R) to 397 °C for the sample containing 20 g/L of RA (R_20RA), confirming that the presence of RA slightly reduces the overall thermal stability of the system (Figure 4b and Table 2). At the same time, a marked increase in the carbonaceous residue at 700 °C is observed, rising from 6.4% for R to 23.1% for R_20RA. This residue cannot be directly correlated with the resin composition; indeed, it is significantly higher than the values expected based on the individual components (R and RA) and their relative proportions (see Table 2), likely due to a more complex degradation mechanism arising from their simultaneous decomposition.

**Table 2:** Thermal Behavior of the Resin Loaded with Increasing RA Content (5, 10, 15, and 20 g/L): Degradation Temperatures (*T*_onset_ and *T*_max_), Residual Content at 700 °C and Corresponding Estimated Values, and *T*_g_ Values Compared to Pure R and RA

sample	*T*_onset_ (°C)^a^	*T*_max_ (°C)^b^	residue at 700 °C (%)	theoretical residues^c^	*T*_g_ (°C)^d^
R	341	405	6.4 ± 0.3		–12.6
RA	225	365	32.0 ± 0.3		
R_5RA	326	404	11.9 ± 0.1	8.9	–6.4
R_10RA	287	402	15.1 ± 0.4	11.0	–1.8
R_15RA	286	400	20.3 ± 0.2	12.7	7.2
R_20RA	274	397	23.1 ± 0.2	14.2	21.7

a Temperature at 5 wt % mass loss.

b Maximum degradation temperature (*T*_max_) determined from the DTG peak (Figure 4b).

c Calculated based on the sample compositions reported in Table 1 and the residue values of the pure components (R and RA); measurements in triplicate.

d Value from the second heating scan of the DSC analysis.

First, RA shows a lower thermal stability^31^ compared to the resin matrix, which may introduce fragments that decompose at lower temperatures and thus anticipate the degradation onset of all resin-based materials. The observed increase in char residue suggests that the decomposition products of RA may influence the pyrolysis behavior of the resin matrix, leading to a higher final residue by promoting the formation of more stable carbonaceous structures. Nevertheless, further investigation would be required to elucidate the underlying mechanisms. Moreover, the possible intermolecular interactions between RA and the resin also play a role in influencing the overall thermal behavior.

DSC analyses from the second heating scan (Figure S6I) reveal a progressive increase in the glass transition temperature (*T*_g_) with increasing RA content. *T*_g_ rises from −12.6 °C for the neat resin to 21.7 °C for the sample containing 20 g/L of RA (Table 2), corresponding to an overall increase of approximately 30 °C. This significant increase indicates a reduction in polymer chain mobility, which may be interpreted as an antiplasticizing effect. Furthermore, the negative deviation from linearity with respect to ideal mixing behavior (Figure S6II) suggests that RA is not acting as an inert diluent but rather influences the relaxation dynamics of the polymer matrix likely due to strong intermolecular interactions. In particular, the phenolic and carboxylic acid groups of RA can establish hydrogen bonding with the ester carbonyl groups in the side chains of the acrylic resin,^48^ (as suggested by FTIR-ATR results), leading to reduced free volume and the formation of a more rigid network structure. Similar increases in *T*_g_ have been reported for other polymer systems incorporating polyphenolic compounds, such as poly­(lactic acid) and poly­(vinyl alcohol) blended with rosmarinic extracts or tea-derived polyphenols,^49,50^ where the small polyphenolic molecules interpenetrate the macromolecular network and form hydrogen-bonded interactions with polymer chains, thereby limiting the segmental mobility of polymer chains.

### 3.3 Characterization of Cotton (C) and Plasma-Activated Cotton (P_C) Coated with Resin (R) and Resin RA-Loaded (R_*x*RA)

Cotton and plasma-activated cotton were coated with the resin samples containing different concentrations of RA. Visual inspection (Figure S7) revealed that, starting from an RA concentration of 15 g/L, visible aggregates formed on the surface (samples d–e and i–l). In untreated samples, these aggregates appear more numerous and more widely distributed (d–e), whereas in plasma-pretreated samples they are less abundant, although still clearly visible (i–l). This may indicate a more homogeneous coating distribution of both the resin and RA on plasma-treated cotton surfaces.

#### 3.3.1 FTIR-ATR Analysis

FTIR-ATR analysis of the coated cotton samples proved to be extremely challenging due to the overlap of diagnostic signals from the different components, namely cotton, resin, and RA, the latter present in increasing amounts.

To facilitate comparison, all FTIR-ATR spectra were normalized to the cotton O–H stretching band at 3333 cm^–1^, as this is the only signal not overlapping with those of the resin and RA, thus enabling a more appropriate and reliable comparison (an example for samples C_R_10RA and P_C_R_10RA is shown in Figure S8). However, at high RA loadings, the absorption bands in the 3650–3200 cm^–1^ region, attributed to the O–H stretching vibrations of phenolic and carboxylic groups, may cause significant interference (see inset in Figure S8).

The FTIR-ATR analysis was therefore focused on the C=O stretching region, as reported for representative samples treated with R_10RA in Figure 5. The numbers following each sample name in the legends indicate the sequential number of points analyzed on the samples, as described in the Experimental section. Figure 5a refers to samples coated with the neat resin, while Figure 5b shows samples coated with RA-loaded resin (R_10RA). In both figures, black lines refer to untreated cotton samples, while red lines correspond to plasma-treated samples.

**Figure 5:**
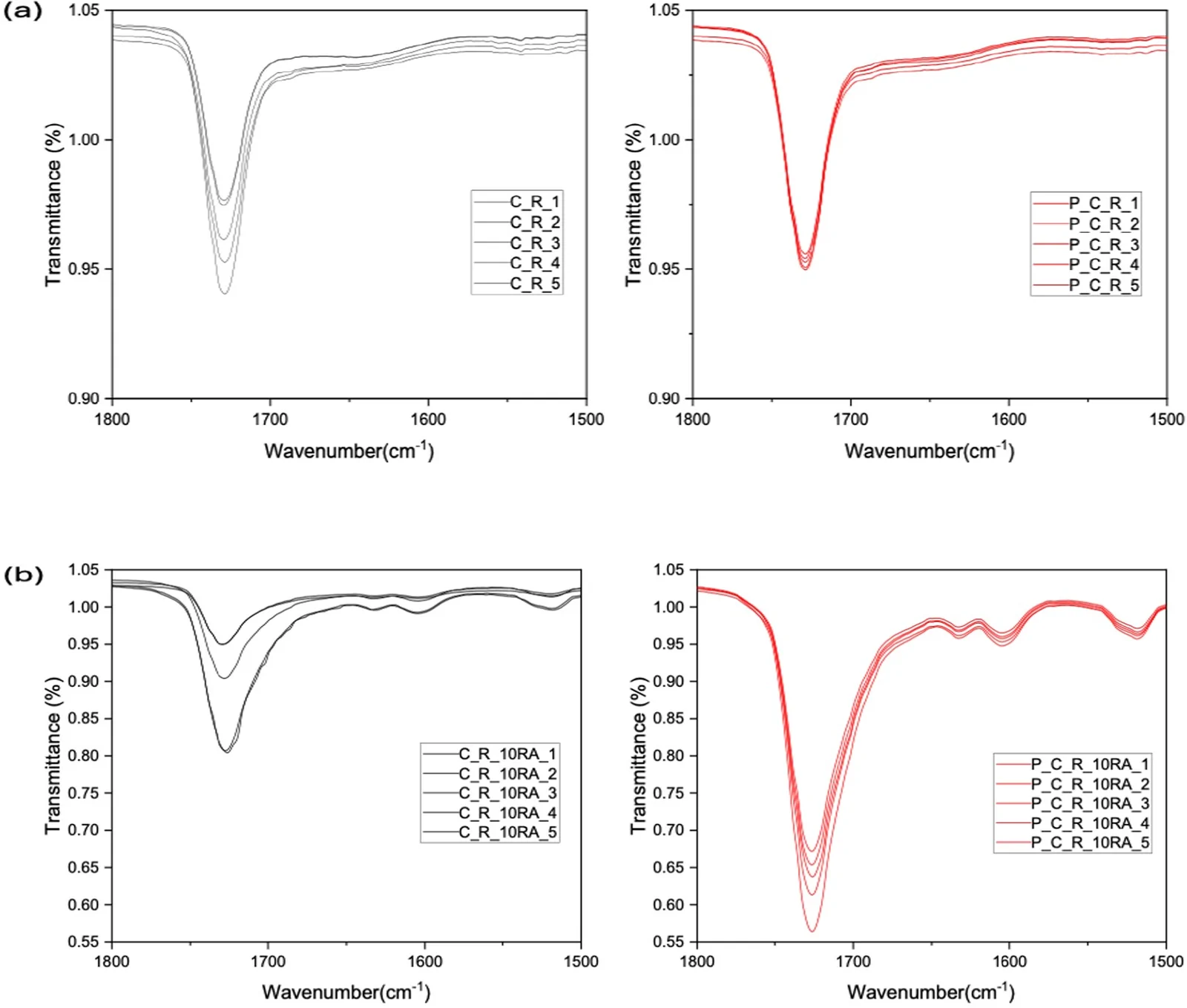
FTIR-ATR spectra of untreated (black) and plasma-treated (red) cotton coated with (a) resin (R); (b) resin containing 10 g/L of RA (R_10RA).

In Figure 5a, the strong band observed at approximately 1730 cm^–1^ is attributed to the C=O (carbonyl) stretching vibration of the ester groups of the resin. In Figure 5b, this band becomes broader and more intense due to the contribution of the C=O group of RA. In addition, as previously discussed, the bands assigned to the C=C stretching vibrations of the conjugated aromatic system and phenolic rings can be clearly evidenced in the region 1630–1520 cm^–1^. What it is really noticeable, in plasma-treated samples is that, the spectra collected at different points are largely superimposable and display more homogeneous signal intensities, indicating a more uniform and homogeneous distribution of the coating over the surface.

As a general trend, although only a qualitative evaluation can be made owing to scattered band intensities, plasma-treated samples appear to contain higher amounts of resin at the surface and, consequently, higher amounts of RA when the resin is more highly loaded with RA (see in particular the Figure 5b). This trend, shown in Figure S9, also for samples treated with resin containing different quantities of RA, can be reliably observed only for samples up to 10 g/L of RA. For higher RA loadings, as already evidenced by visual inspection (Figure S7), the pronounced surface punctual heterogeneity prevents any meaningful comparative FTIR-ATR analysis.

#### 3.3.2 Thermal Characterization (TGA and DSC)

All resin-treated samples exhibit a multistep degradation profile (Figure 6). The first stage, occurring between 40 and 100 °C, is attributed to the evaporation of moisture and results in a weight loss of approximately 4–8% of the initial mass. The second, more significant stage takes place between about 250 and 360 °C and corresponds to the degradation of cellulose, thus being associated with the cotton component. A third degradation step is observed between 360 and 430 °C and is likely related to the resin. However, depending on the RA content, resin degradation may begin at lower temperatures (see Table 2), partially overlapping with the cellulose degradation stage.

**Figure 6:**
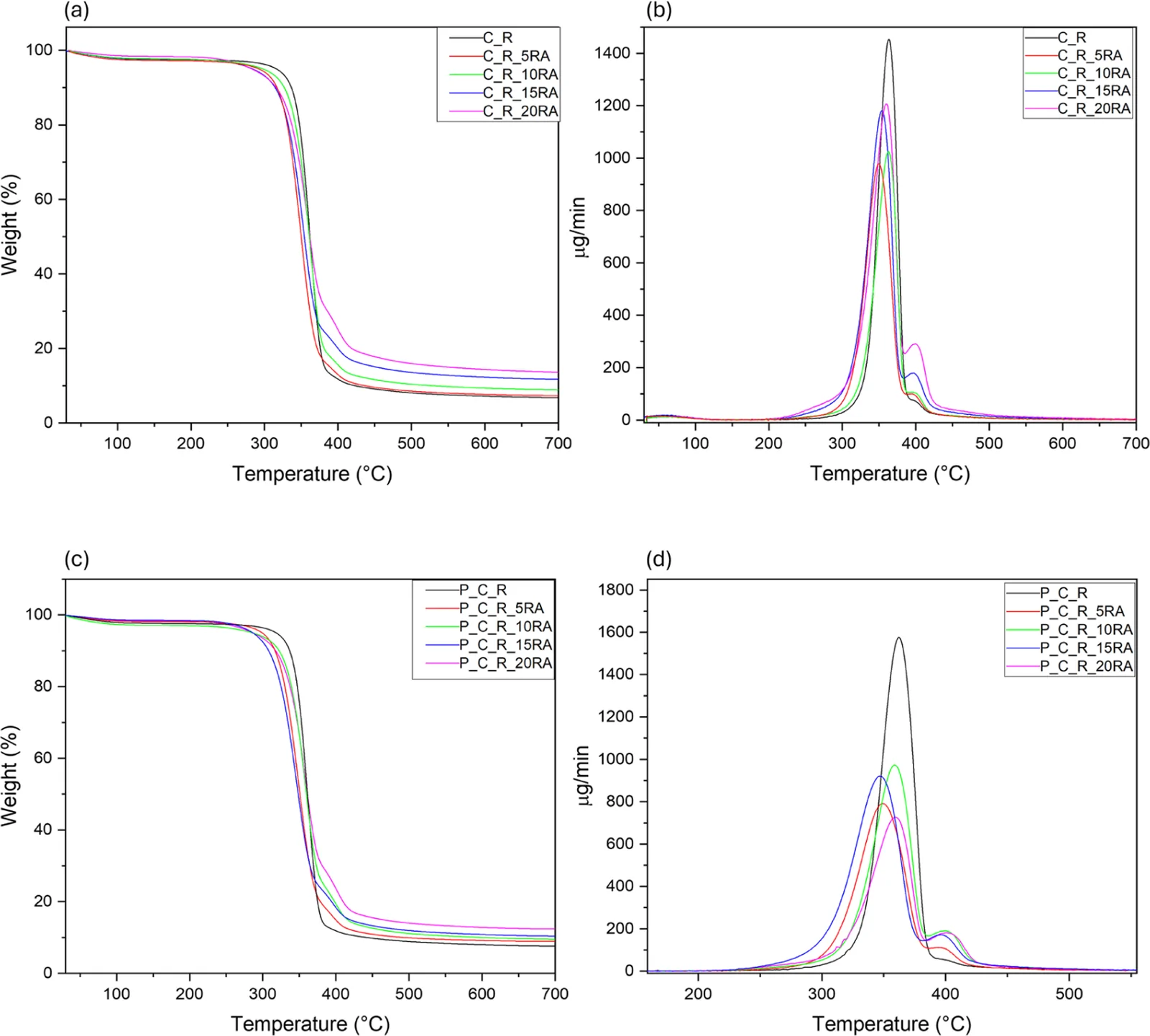
Thermogravimetric analysis (TGA) of cotton and plasma-treated cotton samples coated with resin containing 5, 10, 15, and 20 g/L of RA: (a, c) Weight (%), (b, d) derivative thermogravimetric (DTG).

Observing the curves of the samples without plasma treatment, a slight reduction in thermal stability is evident in the RA-loaded systems. The introduction of RA results in a clear decrease in *T*_onset_, from 343 °C for the sample treated with neat resin (C_R) to approximately 322–330 °C for all RA-loaded samples (Table 3). This indicates that the presence of RA anticipates the onset of the main degradation process. However, no progressive decrease in *T*_onset_ with increasing RA concentration is observed, as the onset temperature remains nearly constant across the 5*–*20 g/L RA concentration range. Plasma treatment does not significantly affect *T*_onset_, since comparable values are obtained for treated and untreated samples at each RA concentration of loaded resin used for the coating. Overall, the changes in degradation temperatures are limited and do not follow a well-defined trend. All TG curves remain very close to each other, suggesting that RA-loaded resins have a limited impact on the thermal stability of cotton, with some variability possibly attributable to partial inhomogeneity of the coating at higher RA loadings. This nonlinear behavior is consistent with the DTG profiles, in which RA-loaded samples exhibit peak broadening and a high-temperature shoulder, indicating overlapping degradation events.

**Table 3:** Thermal Behavior of the Cotton and Plasma-Treated Cotton Samples Coated with Resin Loaded with Increasing RA Content (5, 10, 15, and 20 g/L): Degradation Temperatures (*T*_onset_ and *T*_max_), Residual Content at 700 °C and *T*_g_ Values

sample	residue at 700 °C (%)^a^	*T*_onset_ (°C)^b^	*T*_max_ (°C)^c^	*T*_g_ (°C)^d^
C_R	7.1 ± 0.3	316–343	363	–10.4
P_C_R	7.9 ± 0.2	318–340	363	–9.6
C_R_5RA	7.3 ± 0.1	290–324	349	–8.8
P_C_R_5RA	8.6 ± 0.3	299–323	349	–8.3
C_R_10RA	8.8 ± 0.1	295–330	363	–4.3
P_C_R_10RA	9.7 ± 0.2	285–332	358	–2.4
C_R_15RA	11.0 ± 0.7	284–322	354	–0.5
P_C_R_15RA	12.0 ± 0.3	286–314	345	0.7
C_R_20RA	12.6 ± 1.3	283–325	360	4.0
P_C_R_20RA	13.7 ± 1.6	286–323	360	5.3

a Measurements performed in triplicate; the difference between untreated (C_R_*x*RA) and plasma-treated samples (P_C_R_*x*RA) is statistically significant (Student’s *t* test, *p* < 0.05, *n* = 3) only up to a resin RA loading of 10 g/L. For samples coated with R_15RA and R_20RA, the differences between untreated and plasma-treated cotton are not statistically significant (Student’s *t* test, *p* > 0.05, *n* = 3).

b Value determined as the temperature at 5 wt % mass loss-value calculated from the intersection point of the tangents to the regions before and after degradation.

c Maximum degradation temperature (*Tmax*) determined from the DTG peak (Figure 6b,6d).

d Value obtained from the second heating scan of the DSC analysis.

A key observation concerns the amount of solid residue remaining at the end of thermal degradation. An overall increase in residual mass is observed with increasing RA concentration in the resin. Although some of the numerical data are affected by relatively high standard deviations, (particularly for samples treated with the resins containing high RA loading), and not all pairwise differences are statistically significant (see notes “a” to Table 3), the overall trend remains consistent and can be interpreted in the context of the degradation pathway of the loaded resin discussed above. More importantly, when comparing plasma-treated and untreated cotton samples, the former exhibit a slightly higher residual mass, indicating that plasma treatment enhances the deposition and retention of the RA-loaded resin on the cotton surface (Table 3). This increase can be directly interpreted as an indication of improved coating efficiency promoted by plasma surface activation, in agreement with FTIR-ATR evidence. Plasma treatment likely enhances adhesion and/or the amount of deposited resin, thereby promoting the formation of a greater amount of carbonaceous residue at the end of thermal degradation. This behavior is consistent with previous findings where analyses of plasma/UV-treated coated cotton fabrics with flame retardant agents showed an increase in retention of active agents compared to untreated samples, claiming the occurrence of specific interaction between different functionalities present on plasma treated cotton and coating.^51^

The DSC analysis of coated samples (Table 3) shows that the glass transition temperature (*T*_g_) progressively increases with increasing RA concentration, similarly to what was observed for the neat resin systems containing different amounts of RA. In the untreated samples, *T*_g_ increases from – 10.4 °C (C_R) to 4.0 °C (C_R_20RA), whereas plasma-treated samples exhibit consistently higher *T*g values across all formulations, reaching 5.3 °C at the highest RA concentration.

Although the magnitude of the *T*_g_ increase induced by RA is less pronounced than that observed in bulk resin-rosmarinic acid systems, the overall trend remains consistent. The reduced sensitivity in coated fabrics can be related to interfacial and morphological factors, including partial interactions of RA with cellulose hydroxyl groups^31^ and the formation of RA aggregates at concentrations ≥15 g/L, as evidenced by visual inspection of samples.

Importantly, the increase in *T*_g_ induced by plasma treatment is also observed in the absence of RA, indicating that plasma activation of the cotton substrate independently affects the thermal response of the coated system. This effect can be attributed to improved interfacial adhesion between the acrylic coating and the cellulose fibers, which introduces additional constraints on polymer chain mobility. Overall, the DSC results indicate that RA contributes to an intrinsic stiffening of the acrylic matrix, while plasma treatment provides an additional, RA-independent interfacial constraint that further increases *T*_g_ in coated cotton systems.

#### 3.3.3 Morphological Analysis (SEM)

Figure 7 shows SEM micrographs of untreated and plasma-treated cotton samples coated with the neat resin (R) and with the resin containing 10 g/L of RA (R_10RA), collected at different magnifications (100×, 500×, and 4000×). In all cases, the presence of the coating is evident, indicating that the resin-based formulations effectively cover the fiber surface after the applied dip-coating and curing steps. However, clear morphological differences can be observed between untreated and plasma-treated substrates.

**Figure 7:**
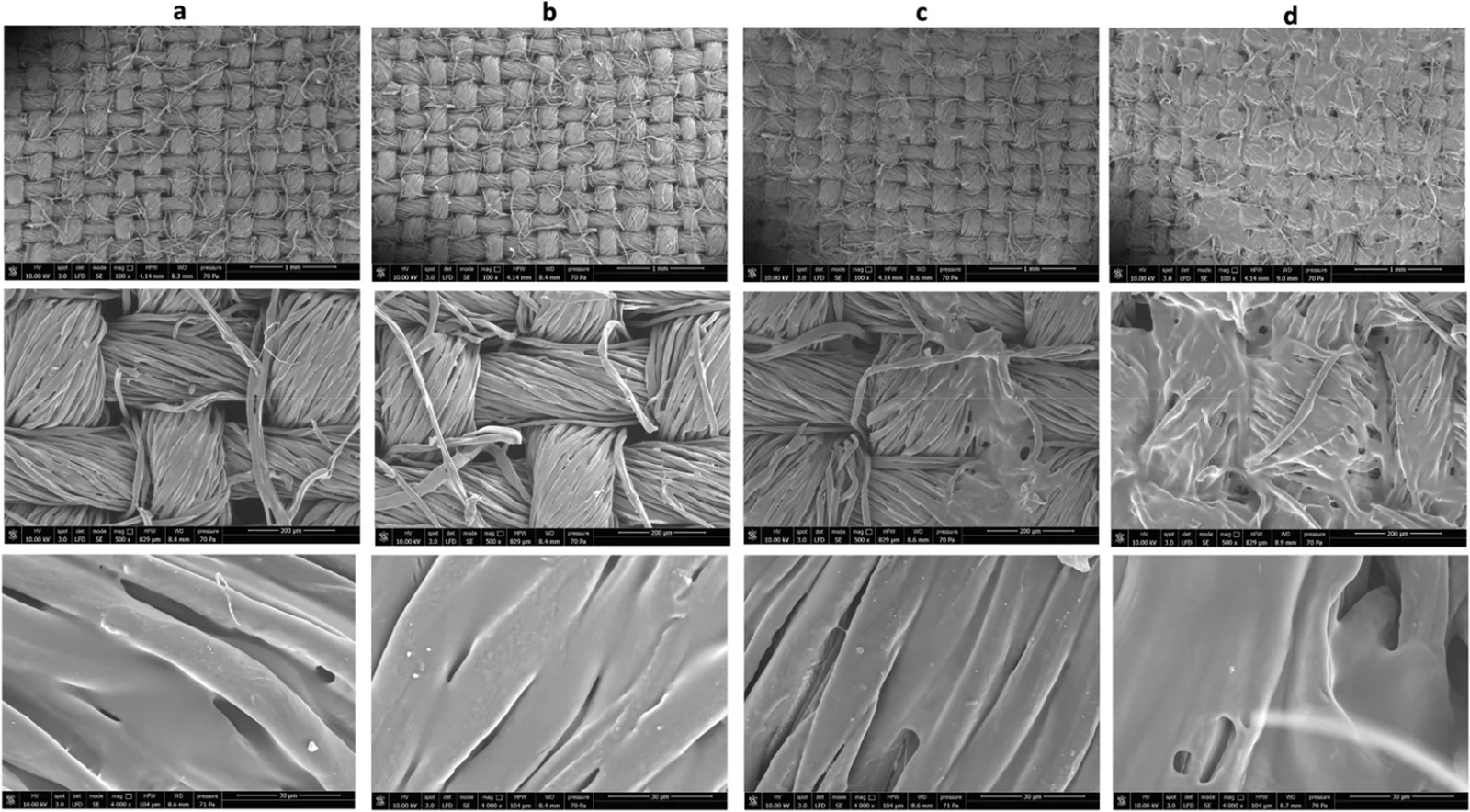
SEM images at different magnifications (100×, 500× and 4000×) of cotton and plasma-treated cotton samples coated with R and R_10RA. Specifically: (a) C_R; (b) P_C_R; (c) C_R_10RA; (d) P_C_R_10RA.

In all samples, the coating is clearly visible and forms a continuous layer that effectively covers the fiber surfaces, regardless of plasma treatment. However, in plasma-treated samples, the coating appears more homogeneous and tends to bridge the gaps between adjacent fibers, resulting in a more uniform and continuous surface morphology. Although it cannot conclusively support the hypothesis that plasma treatment increases the amount of deposited resin or significantly improves the overall coverage, the enhanced surface uniformity (particularly for samples treated with the resin containing 10 g/L of RA) is consistent with both visual inspection and FTIR-ATR analysis.

In the untreated coated samples, the coating appears distributed over the fiber surface but with a less regular morphology, and local variations in surface texture can be distinguished. By contrast, in plasma-treated samples the deposited layer appears more continuous and homogeneous, with a more uniform coverage of the fiber contours. This effect is particularly evident in the samples coated with the formulation containing RA, in which the plasma-treated substrate shows a smoother and more evenly distributed coating phase. In addition, the coating in these samples tends to partially bridge the interfiber spaces, suggesting a more efficient spreading of the liquid formulation before curing and the formation of a more continuous interfacial layer after thermal treatment.

These observations are consistent with the surface modifications induced by oxygen plasma treatment discussed above. The introduction of oxygen-containing functionalities and the increase in surface roughness are expected to raise the surface energy of cotton and improve wetting by the resin formulation. As a consequence, plasma activation can promote a more regular distribution of the coating over the fiber surface and improve interfacial contact between the acrylic matrix and the cellulosic substrate. In this respect, the SEM results support the FTIR-ATR evidence indicating a more homogeneous surface coverage in plasma-treated samples.

The comparison between the neat resin and the RA-loaded resin also provides useful information. At the intermediate loading examined here (10 g/L), no large isolated crystalline domains are observed by SEM, and the coating retains an overall continuous character. This suggests that, at this concentration, RA can still be incorporated within the resin layer without generating the marked surface heterogeneity already detected by visual inspection for the more highly loaded formulations. Therefore, the R_10RA sample can be regarded as representative of a condition in which the bioactive compound is present while the coating morphology remains sufficiently regular to allow meaningful comparison between untreated and plasma-treated cotton.

At the same time, SEM does not provide direct evidence of the actual amount of resin deposited, nor of the molecular-scale distribution of RA within the coating. Therefore, the present images should be interpreted primarily as qualitative evidence of improved coating continuity and uniformity after plasma pretreatment, rather than as direct proof of higher coating mass or deeper penetration into the textile structure. Nevertheless, when considered together with the visual inspection, FTIR-ATR results, and antibacterial tests, the SEM observations support the conclusion that plasma pretreatment favors a more regular organization of the functional layer on cotton, which is likely beneficial for the reproducibility and effectiveness of the antibacterial coating.

#### 3.3.4 Antimicrobial Tests

The antibacterial results reported in Table 4 show that the activity of the RA-containing coatings is strongly dependent on both the bacterial strain and the formulation conditions. A clear difference emerges between the responses of *S. aureus* and *E. coli*. In the case of *S. aureus*, the coatings already exhibit marked activity at the lowest RA loading, with bacterial reduction increasing from 85.3% for C_R_5RA to 99.0% for P_C_R_5RA. From 10 g/L upward, all formulations reach complete or nearly complete reduction of the Gram-positive strain, indicating that, under the adopted test conditions, *S. aureus* is highly susceptible to the RA-based coating system.

**Table 4:** Antibacterial Activity of Cotton Coated with R_*x*RA against *S. aureus* (Gram-Positive) and *E. coli* (Gram-Negative)

sample	RA resin loading (g/L)	*S. aureus*	*E. coli*
RA	–	100.0 ± 0.0	
C_R_5RA	5	85.3 ± 0.0	13.2 ± 8.9
P_C_R_5RA	5	99.0 ± 1.4	0
C_R_10RA	10	100.0 ± 0.0	78.0 ± 4.5
P_C_R_10RA	10	100.0 ± 0.0	59.1 ± 2.7
C_R_15RA	15	100.0 ± 0.0	85.5 ± 0.9
P_C_R_15RA	15	100.0 ± 0.0	100.0 ± 0.0
P_C_R_20RA	20	100.0 ± 0.0	98.7 ± 0.0

A different trend is observed for *E. coli*, for which the antibacterial response is much more sensitive to RA loading and to the surface condition of the substrate. At 5 g/L, the activity is negligible or absent, whereas at 10 g/L only partial bacterial reduction is achieved. Near complete or complete activity is reached only at higher RA contents, namely at 15 and 20 g/L. This behavior indicates that the response against *E. coli* follows a threshold-like trend rather than a simple proportional increase with RA content, suggesting that a minimum effective surface availability of the active compound is required before a strong antibacterial effect can be established against the Gram-negative strain.

The antibacterial activity of the coated samples is primarily governed by the presence and surface availability of RA within the resin layer. Since all fabrics were dip-coated with either neat resin or RA-loaded resin, plasma treatment does not directly introduce any intrinsic antibacterial functionality. Instead, its role should be regarded as indirect, as a surface-activation step that modifies the physicochemical properties of cotton prior to coating deposition. In particular, oxygen plasma treatment increases surface energy and roughness, thereby improving wetting, adhesion, and overall distribution of the resin-based layer on the fiber surface. These effects can influence the antibacterial response by modulating the effective exposure and accessibility of RA at the fiber/liquid interface during the test, rather than by contributing directly to bacterial inactivation. In this sense, differences observed between untreated and plasma-treated samples should be interpreted in terms of coating organization and interfacial effects, which may affect the local availability of the active compound.

Accordingly, plasma treatment should not be considered an independent antibacterial factor, but rather a process that can indirectly affect performance depending on the coating composition and RA loading.

When these results are considered together with the morphological and spectroscopic evidence discussed above, a coherent picture emerges. Plasma pretreatment appears to favor a more homogeneous coating distribution on cotton, but improved homogeneity does not necessarily translate into higher antibacterial activity at low RA loading, where the amount of accessible active compound may still remain below the threshold required for efficient inhibition of *E. coli*. At intermediate loading, particularly at 15 g/L, the improved distribution promoted by plasma treatment appears to become advantageous, enabling full broad-spectrum activity. At the highest loading, by contrast, the antibacterial effect is already very high even without plasma pretreatment, suggesting that the amount of RA becomes the dominant factor and can, at least in part, compensate for differences in coating organization.

Overall, the data indicate that RA is an effective metal-free antibacterial additive for cotton coatings, with particularly high activity against *S. aureus*. The behavior against *E. coli* is more demanding and highlights the importance of achieving not only sufficient RA loading, but also an appropriate balance between coating continuity, surface availability of the active phase, and possible aggregation phenomena. From this perspective, the plasma-treated sample containing 15 g/L RA represents the most balanced formulation of the present series, since it provides complete antibacterial activity against both strains without requiring the highest RA concentration.

## 4 Conclusion

In this work, cotton fabrics were functionalized with a commercial biobased acrylic resin containing increasing amounts of RA, and the effect of oxygen plasma pretreatment on the organization of the coating, thermal behavior, and antibacterial response was investigated. The results show that plasma treatment effectively modifies the cotton surface and promotes a more homogeneous distribution of the resin-based coating, as supported by visual inspection, FTIR-ATR analysis, and SEM observations. Plasma-treated samples also generally exhibited slightly higher residual mass after thermogravimetric analysis, consistent with an improved retention of the deposited functional layer on the fiber surface.

At the formulation level, the incorporation of RA influenced the thermal behavior of the resin, causing a moderate decrease in thermal stability and a progressive increase in the glass transition temperature. This behavior is consistent with intermolecular interactions between the polyphenolic compound and the acrylic matrix, which reduce chain mobility. In the coated fabrics, the effect of RA on thermal stability was less pronounced, but the increase in residual mass and glass transition temperature remained consistent with the presence of the active compound within the coating. At the same time, the formation of visible aggregates at RA concentrations of 15 g/L and above indicates that increasing the loading not simply improves the system, but also introduces morphological heterogeneity that must be taken into account when interpreting the functional response.

From a biological viewpoint, the RA-based coatings showed high antibacterial activity against *S. aureus*, whereas the response against *E. coli* was more dependent on RA loading and coating organization. In particular, complete antibacterial activity against both strains was achieved with the plasma-treated sample containing 15 g/L RA, whereas lower loadings were less effective against the Gram-negative strain. These results indicate that RA can act as an effective metal-free antibacterial component in resin-coated cotton, although its performance depends not only on its concentration but also on its distribution and retention on the textile surface.

Overall, the present results support the feasibility of combining oxygen-plasma pretreatment with a biobased acrylic resin containing RA to obtain metal-free antibacterial coatings on cotton. However, the study should be regarded as a proof of concept, and further work is required to assess washing durability, possible leaching of the active compound, long-term stability, and the reproducibility of the coating add-on. These aspects will be essential to establish the practical potential of this metal-free finishing strategy for textile applications.

## Supplementary Material



## Notes

Schematic representation of the plasma system used for surface treatment (Figure S1); (I) points analyzed on each side (A) and (B) of the cotton sample prior to plasma treatment, (II) FTIR-ATR spectra of the two sides of the cotton sample, comparing side A and side B, the characteristic spectral features discussed in the text are indicated in the figure (Figures S2); FTIR-ATR spectra of untreated and plasma-treated cotton: (a) full spectra; (b) magnified view of the selected region (3000–1400 cm^–1^) (Figure S3); XPS survey spectra of untreated cotton (C) and plasma-treated cotton (P_C) (Figure S4); thermogravimetric (TG) and derivative thermogravimetric (DTG) curves of untreated and plasma-treated cotton, solid line: mass loss (%); dashed line: rate of mass loss (Figure S5); (I) 2nd heating runs evidencing the *T*_g_ of resin and RA-loaded resin; (II) *T*_g_ values as function of RA content (Figure S6); pictures of prepared samples: (a) untreated, coated with resin; (b–e) untreated, coated with resin containing 5, 10, 15, and 20 g/L of RA, respectively; (f) plasma-treated, coated with resin; (g–l) plasma-treated, coated with resin containing 5, 10, 15, and 20 g/L of RA, respectively (Figure S7); FTIR-ATR spectra normalized to the cotton O–H stretching band at 3333 cm^–1^, of untreated (black lines) and plasma-treated (red lines) cotton-coated samples with a resin containing 10 g/L rosmarinic acid, inset report the region between 4000 and 2500 cm^–1^, highlighting the band used to normalize the spectra (Figure S8); FTIR-ATR spectra normalized to the cotton O–H stretching band at 3333 cm^–1^, of untreated (straight lines) and plasma-treated (dashed lines) cotton-coated samples with resin (R) and resin loaded with RA (R_RA5 and R_RA10). On the right the enlargement of the region between 1800 and 1500 cm^–1^, highlighting the C=O stretching vibrations (Figure S9) (PDF)
