# Supplementary material for: Antibacterial Coatings on Cotton Fabrics Based on Rosmarinic Acid-Loaded Bio-Based Resin and Oxygen-Plasma Surface Pretreatment
Source: ACS Omega. 2026 Sep 23;11(39):59281–95. doi: 10.1021/acsomega.6c05181 (PMC13647840; doi:10.1021/acsomega.6c05181)

Antibacterial Coatings on Cotton Fabrics Based on Rosmarinic Acid-Loaded Bio-Based Resin and Oxygen-Plasma Surface Pretreatment

Norma Mallegni[1]*, Serena Facchiano[2], Serena Coiai[1], Francesca Cicogna[1], Roberto Spiniello[1], Randa Ishak[3], Giuseppe Rosace[4], Valentina Trovato[4], Claudia Vineis[2], Stefano Caporali[5] and Elisa Passaglia[1]

[1]National Research Council-Institute for the Chemistry of OrganoMetallic Compounds (CNR-ICCOM), SS Pisa, Via Moruzzi 1, 56124 Pisa, Italy;

[2]National Research Council- Institute of Intelligent Industrial Technologies and Systems for Advanced Manufacturing (CNR-STIIMA) Corso Giuseppe Pella 16, 13900, Biella, Italy;

[3]Department of Civil and Industrial Engineering, University of Pisa, Largo L. Lazzarino 1, 56122 Pisa, Italy;

[4]Department of Engineering and Applied Sciences, University of Bergamo, Viale Marconi 5, 24044 Dalmine (BG), Italy

[5]Department of Industrial Engineering, DIEF, University of Florence, Via S. Marta 3, 50139 Florence, Italy

Corresponding authors norma.mallegni@cnr.it

Figure S1 Schematic representation of the plasma system used for surface treatment.

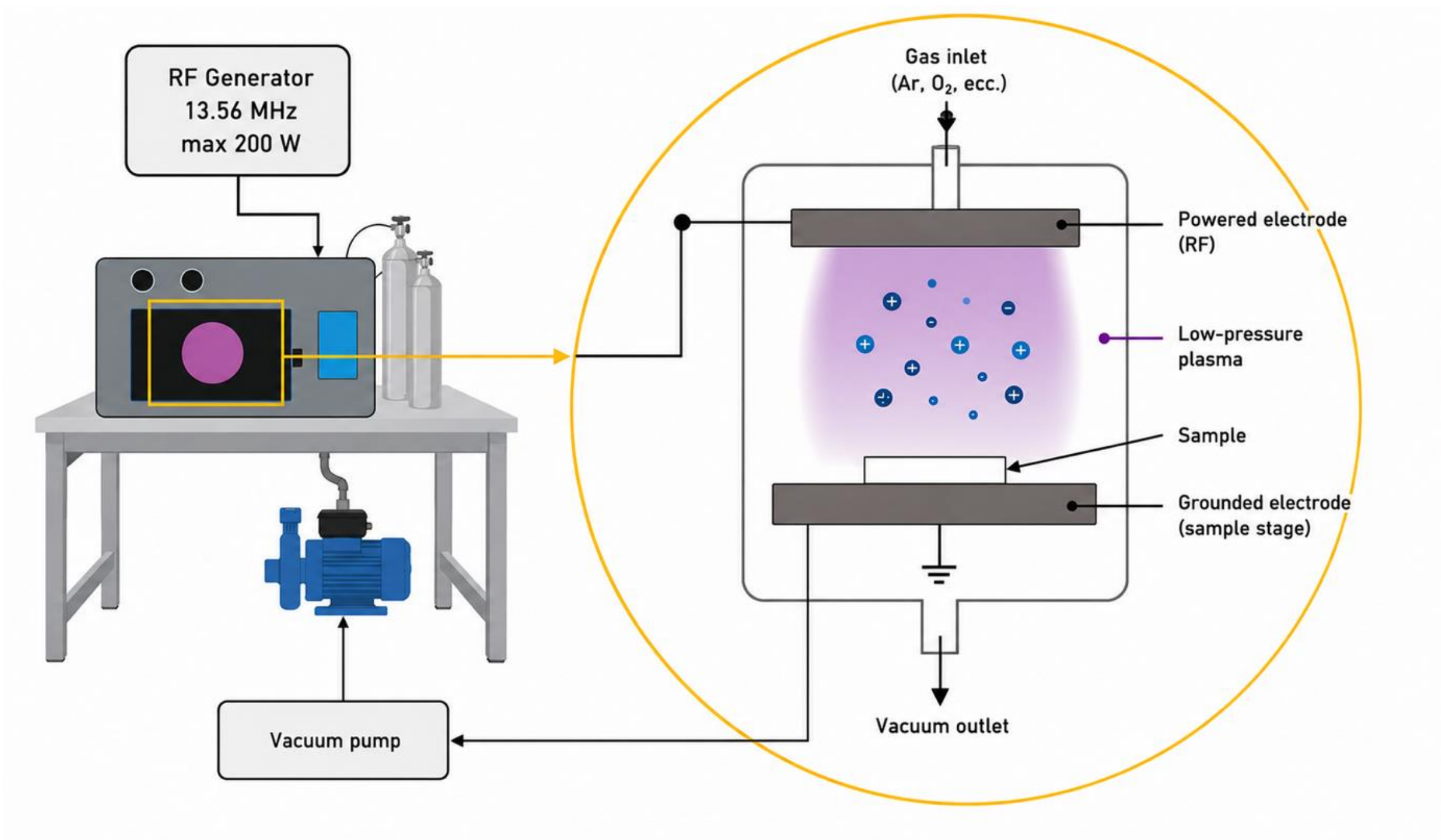

Figure S2: (I) Points analyzed on each side (A) and (B) of the cotton sample prior to plasma treatment. (II) FTIR-ATR spectra of the two sides of the cotton sample, comparing side A and side B. The characteristic spectral features discussed in the text are indicated in the figure.

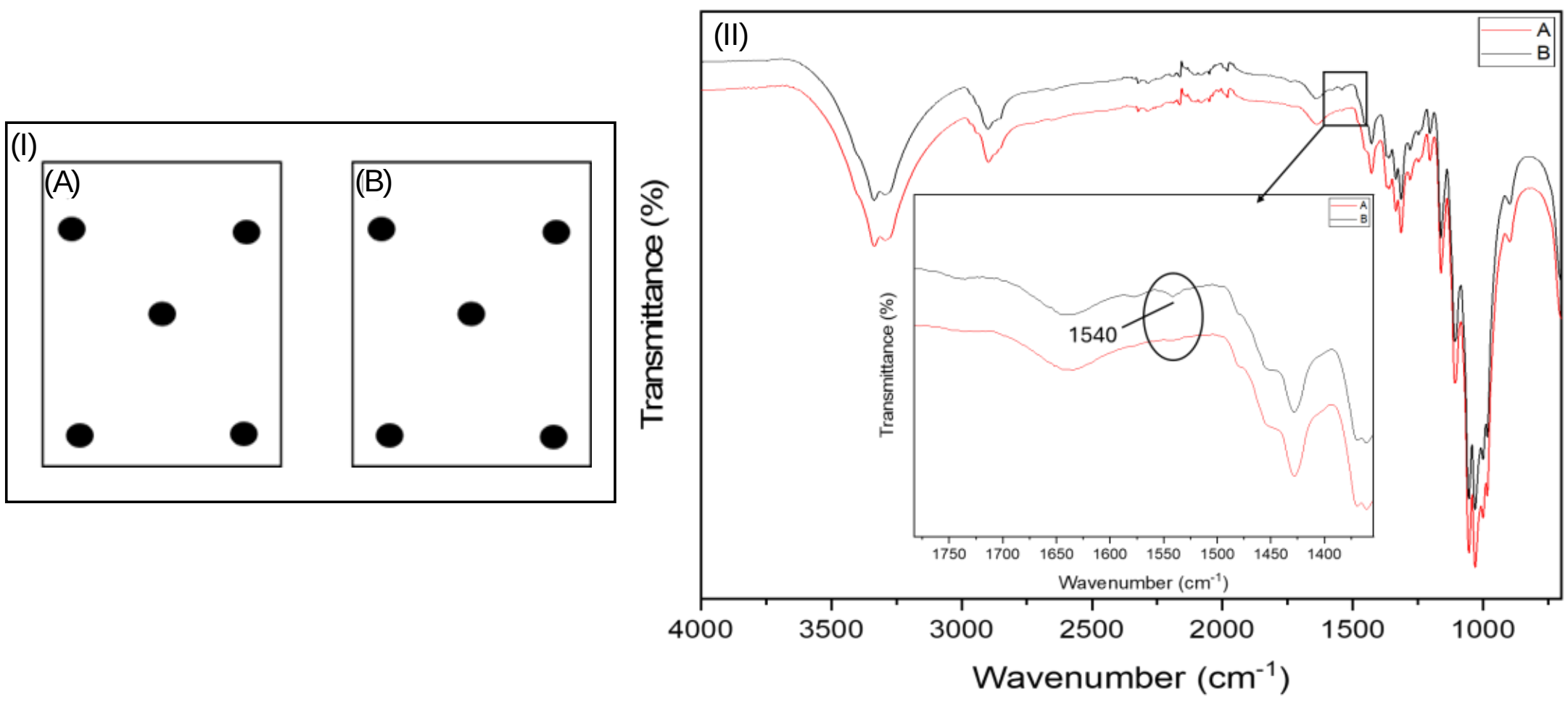

Figure S3. FTIR-ATR spectra of untreated and plasma-treated cotton: (a) full spectra; (b) magnified view of the selected region (3000–1400 cm^{-1}).

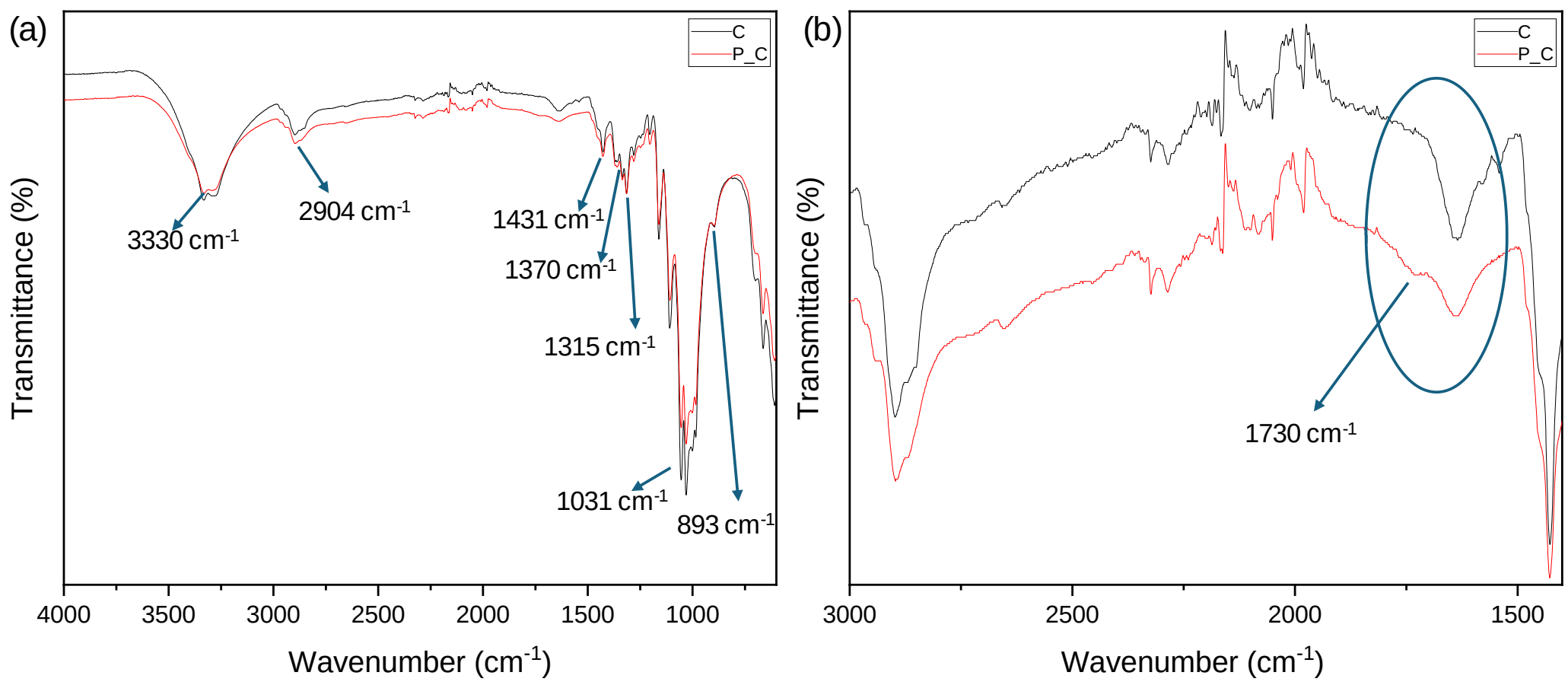

Figure S4: XPS survey spectra of untreated cotton (C) and plasma-treated cotton (P_C).

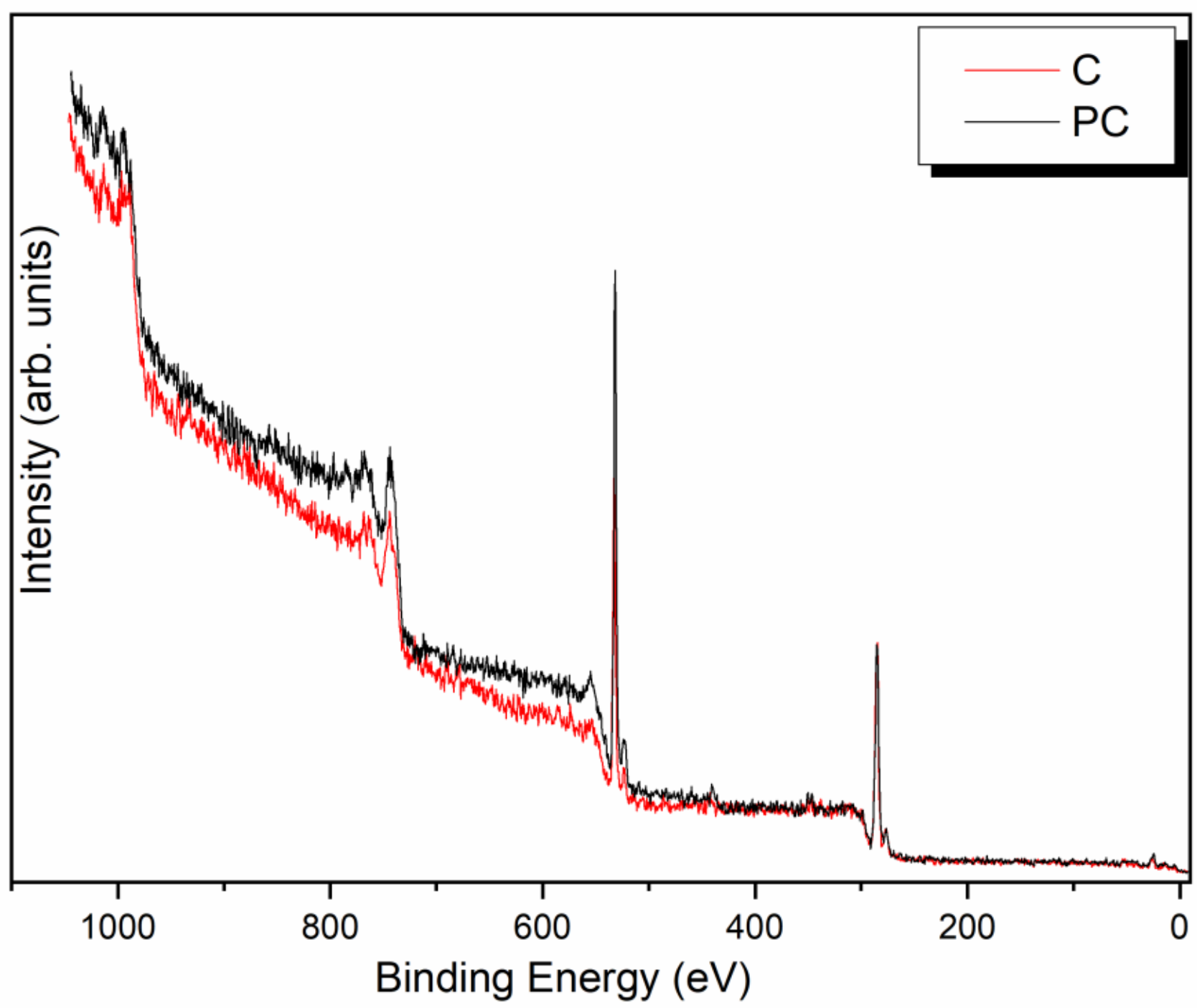

Figure S5: Thermogravimetric (TG) and derivative thermogravimetric (DTG) curves of untreated and plasma-treated cotton. Solid line: mass loss (%); dashed line: rate of mass loss

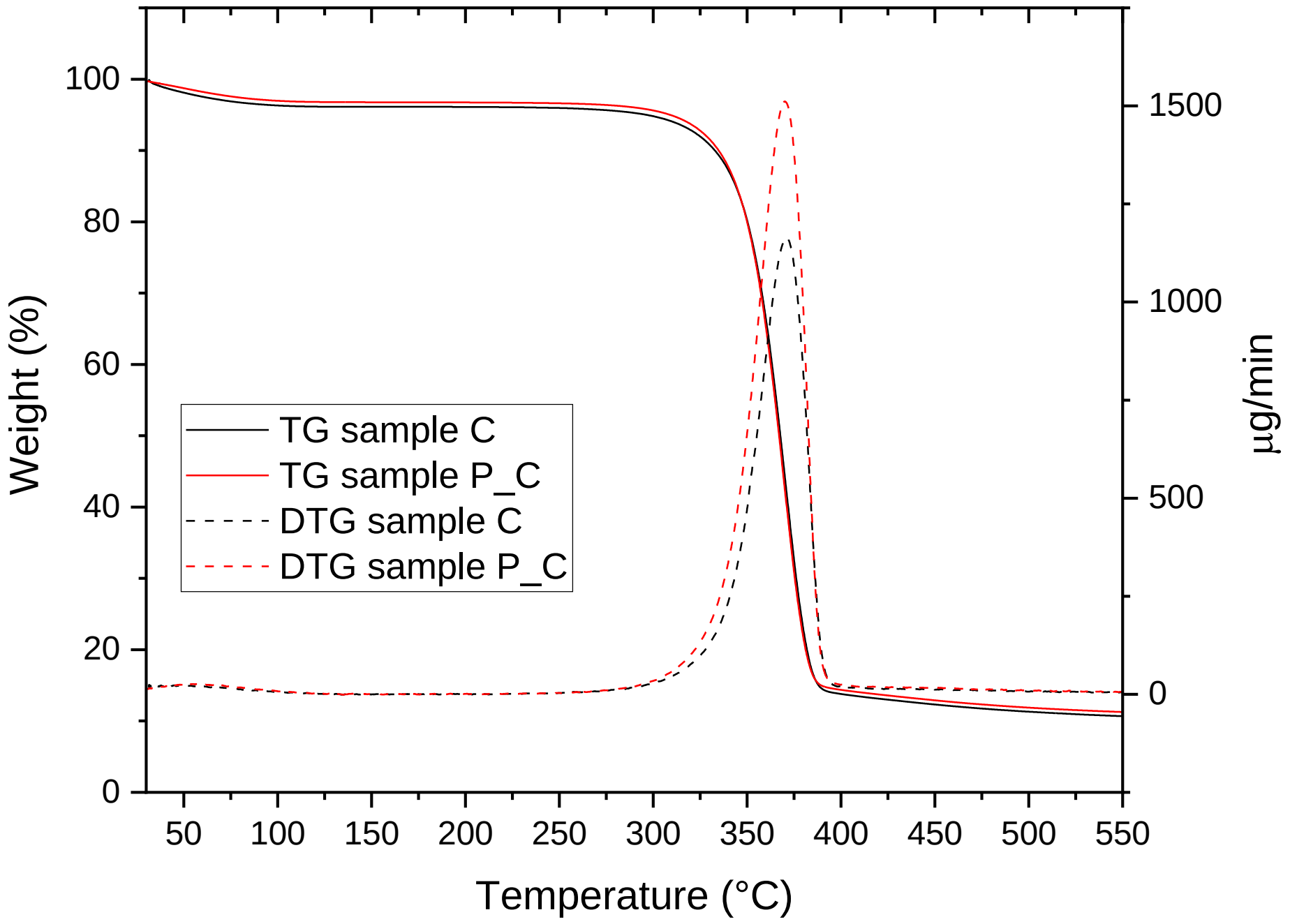

Figure S6: (I) 2nd heating runs evidencing the *T*g of resin and RA-loaded resin; (II) *T*g values as function of RA content. R_xRA refers to resin formulations prepared with x g L^{-1} of rosmarinic acid in the starting coating solution.

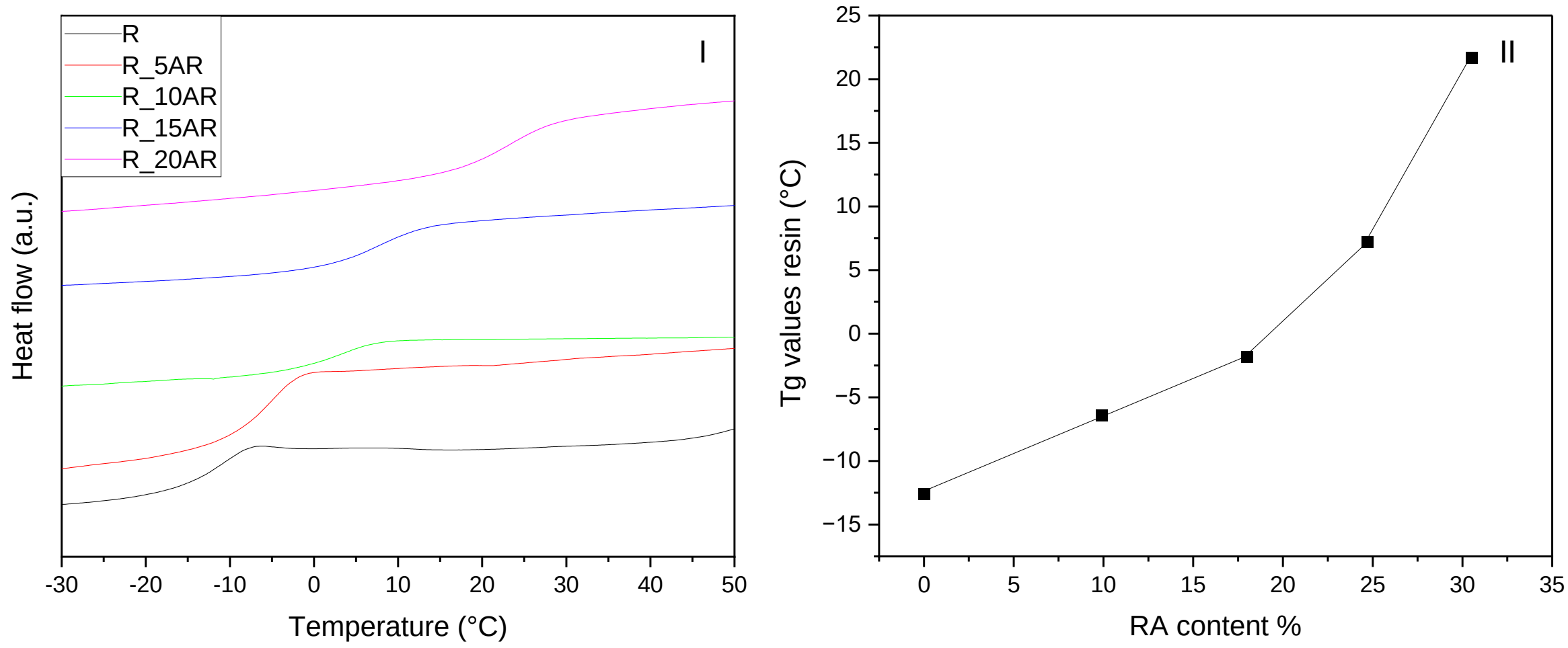

Figure S7 Pictures of prepared samples: (a) untreated, coated with resin; (b–e) untreated, coated with resin containing 5, 10, 15, and 20 g/L of RA, respectively; (f) plasma-treated, coated with resin; (g–l) plasma-treated, coated with resin containing 5, 10, 15 and 20 g/L of RA, respectively

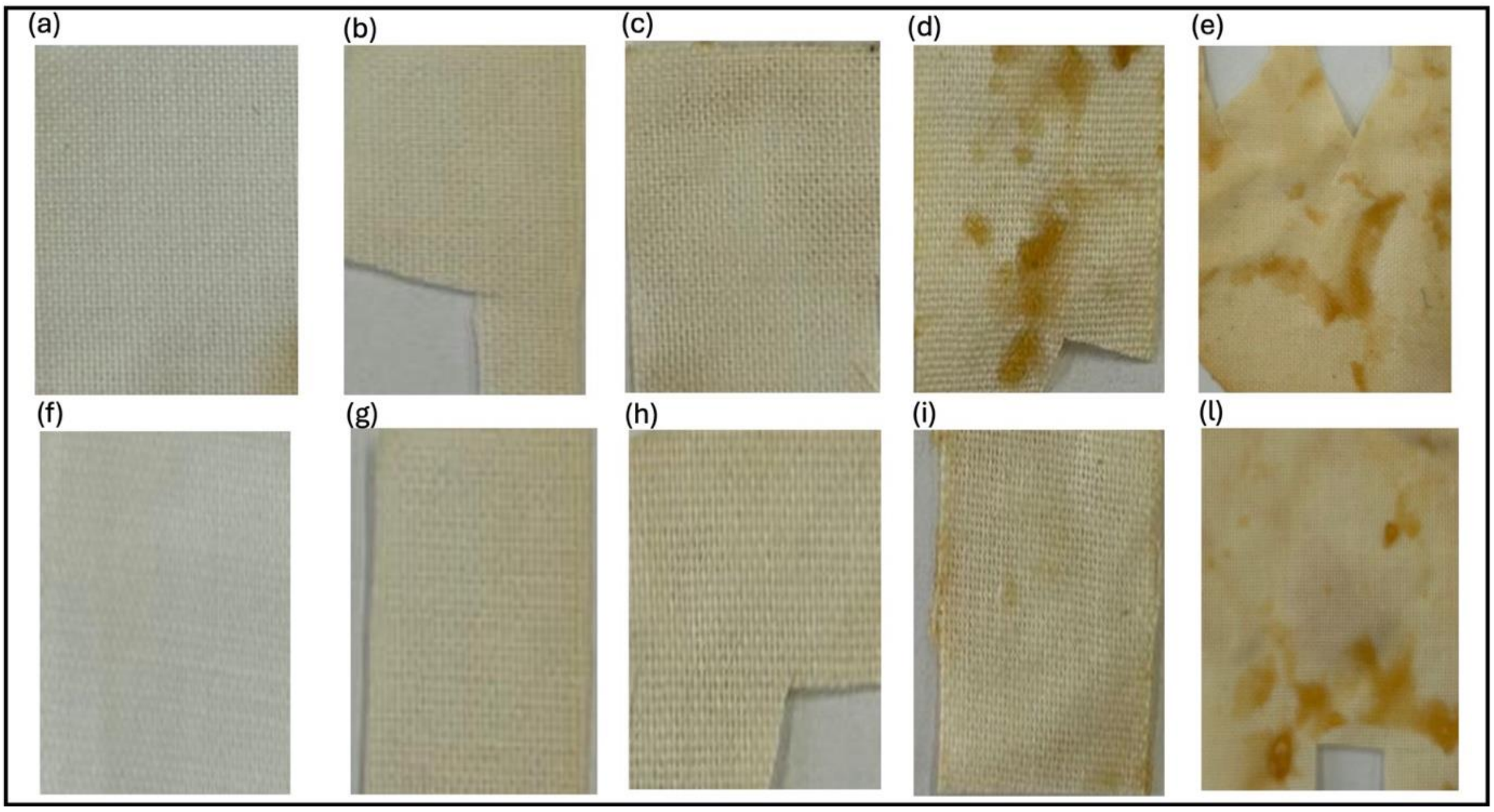

Figure S8: FTIR-ATR spectra normalized to the cotton O–H stretching band at 3333 cm⁻¹, of untreated (black lines) and plasma-treated (red lines) cotton-coated samples with a resin containing 10 g/L rosmarinic acid. Inset report the region between 4000 and 2500 cm⁻1, highlighting the band used to normalize the spectra.

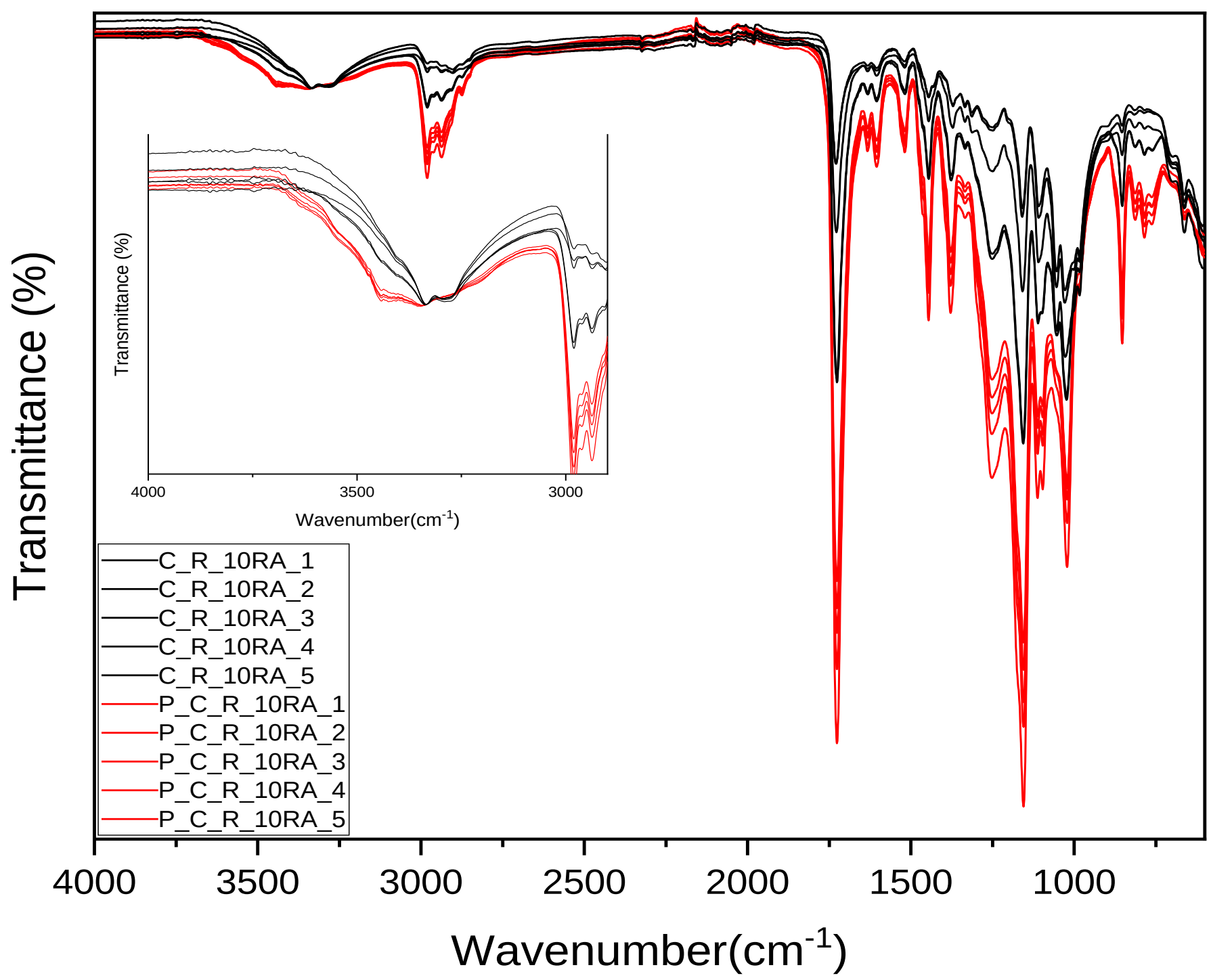

Figure S9: FTIR-ATR spectra normalized to the cotton O–H stretching band at 3333 cm^{-1}, of untreated (straight lines) and plasma-treated (dashed lines) cotton-coated samples with resin (R) and resin loaded with RA (R_RA5 and R_RA10). On the right the enlargement of the region between 1800 and 1500 cm^{-1}, highlighting the C=O stretching vibrations

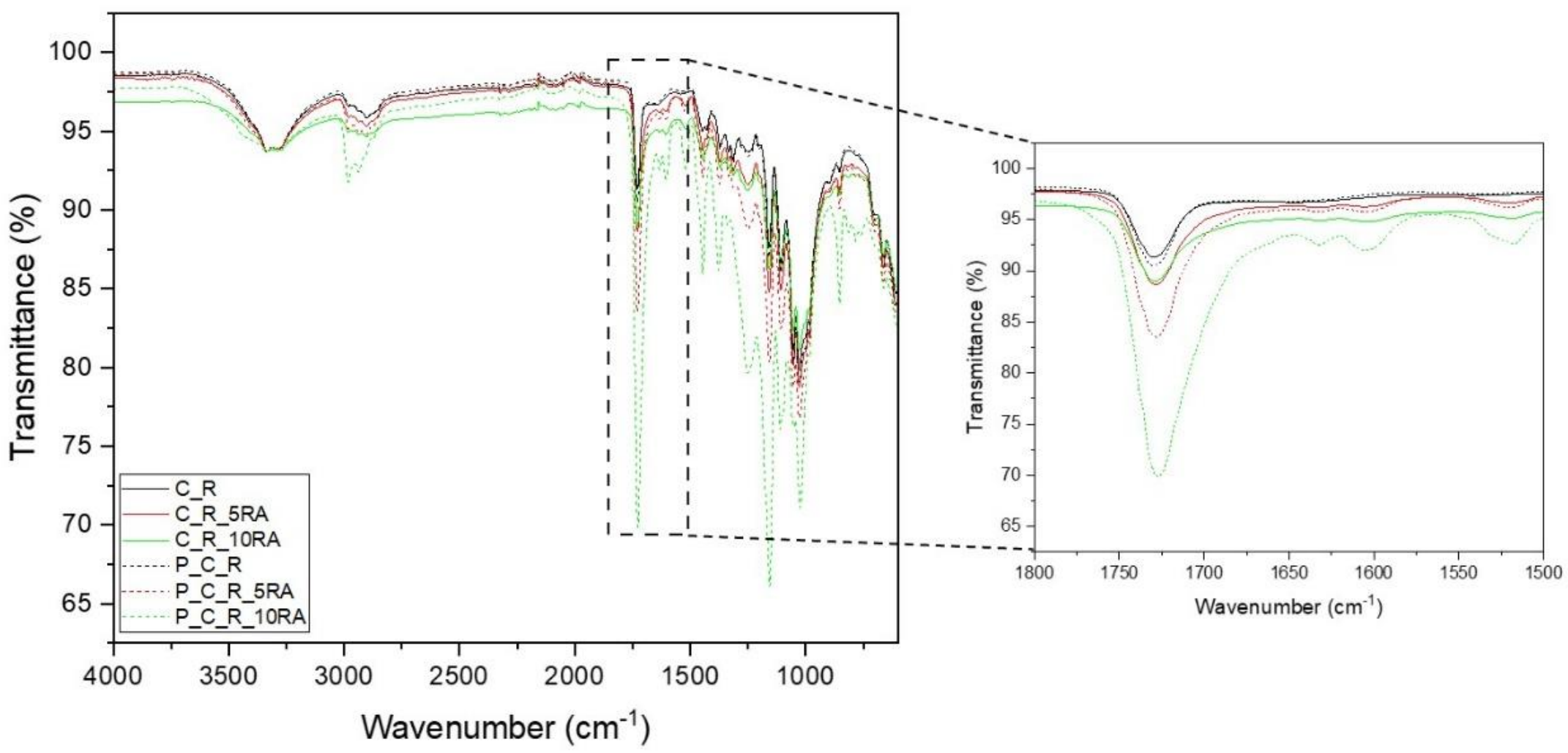

Supplement: Supplementary file 1 [file ao6c05181_si_001.pdf]
